# Pharmacological Inhibition of c‐Jun N‐Terminal Kinase Activity Exacerbates Liver Damage in *Schistosoma mansoni* Infected Mice

**DOI:** 10.1111/liv.70260

**Published:** 2025-08-02

**Authors:** Frederik Stettler, Lukas Knedla, Maximilian Hagen, Verena von Bülow, Heike Müller, Annette Tschuschner, Daniel Zahner, Simone Haeberlein, Max F. Moescheid, Anita Windhorst, Monika Burg‐Roderfeld, Dieter Glebe, Bernardo Pereira Moreira, Céline Lesieur, Andreas Schmid, Franco H. Falcone, Christoph G. Grevelding, Elke Roeb, Martin Roderfeld

**Affiliations:** ^1^ Department of Gastroenterology Justus Liebig University Giessen Giessen Germany; ^2^ Central Laboratory Animal Facility Justus Liebig University Giessen Giessen Germany; ^3^ Institute of Parasitology, BFS Justus Liebig University Giessen Giessen Germany; ^4^ Institute of Medical Informatics Justus Liebig University Giessen Giessen Germany; ^5^ Hochschulen Fresenius GmbH, University of Applied Sciences Idstein Germany; ^6^ Institute of Medical Virology, National Reference Center for Hepatitis B Viruses and Hepatitis D Viruses, German Center for Infection Research (DZIF, Partner Site Giessen‐Marburg‐Langen), Justus Liebig University Giessen Germany; ^7^ Polytech Angers, Université D'angers Angers France; ^8^ Department of Internal Medicine III Giessen University Hospital Giessen Germany

**Keywords:** fibrosis, glycogen exhaustion, parasite, schistosomiasis, SP600125

## Abstract

**Background and Aims:**

Schistosomiasis is a neglected tropical disease affecting more than 250 million people worldwide. Eggs of the parasitic helminth *S. mansoni* cause major morbidity in the liver, spleen and intestine. Of note, egg‐released soluble antigens (SEA) induce the transcription factor c‐Jun in hepatocytes, promoting hepatocellular cell cycle activity, proliferation and apoptosis. In this study, we analysed the hepatic effect of pharmacological inhibition of c‐Jun N‐terminal kinase (JNK) after infection with *S. mansoni* . The JNK inhibitor SP600125 was chosen because it had no effect on schistosome viability.

**Methods:**

Eight‐week‐old male mice were infected with 100 cercariae (♂ + ♀) and 6 weeks later treated with SP600125 via a subcutaneously implanted osmotic pump over 3 weeks. Hepatic damage, inflammation, fibrosis and metabolic aspects were analysed in liver and spleen tissue as well as in serum samples.

**Results:**

JNK inhibitor‐treated mice infected with *S. mansoni* showed a parasite‐induced elevation of serum aminotransferases. Hepatic inflammation, the activation of hepatic stellate cells and metabolic exhaustion were observed in infected control mice. Additional SP600125 application almost doubled enhanced transaminases, hepatic cytokine expression, inflammation, necrosis, as well as HSC activation, and decreased glycogen stores to a minimum.

**Conclusions:**

Our findings suggest a protective role of JNK/c‐Jun‐signalling in hepatic inflammation, hepatic stellate cell activation, and metabolic exhaustion during *S. mansoni* infection.

**Summary**

- Previous studies demonstrated a pivotal role of JNK/c‐Jun signalling in cell survival, cell death and tumourigenesis.
- The current study demonstrates that JNK/c‐Jun signalling is essential for protection against pronounced *S. mansoni* ‐induced liver damage.
- Exhaustion of hepatic carbohydrate storage, activation of hepatic stellate cells, and potentiation of hepatic inflammation may contribute to the aggravation of liver damage in *S. mansoni* ‐infected mice under the influence of a pharmacological JNK inhibition.

**Abbreviations**
AP‐1: activator protein 1
hJNK: human JNK
HSC: hepatic stellate cell
IPSE: interleukin‐4‐inducing principle of *S. mansoni* eggs
JNK: c‐Jun N‐terminal kinase
KLIFS: kinase‐ligand interaction fingerprints and structures

*S. mansoni*

:
*Schistosoma mansoni*

SEA: soluble egg antigen
SmJNK:
*S. mansoni* JNK
SP600125: JNK inhibitor
TSA: thermal shift assay

## 1 Introduction

Schistosomiasis, one of the most important parasitic infections, is responsible for public health problems in more than 78 countries. According to the World Health Organisation (WHO), more than 250 million people required preventative treatment in 2021 [1]. Due to its high prevalence in developing regions such as the Middle East, South America, Southeast Asia and Sub‐Saharan Africa, schistosomiasis is recognised as a neglected tropical disease (NTD) [2]. Globalisation, climate change, and the resulting expansion of the intermediate host's habitat into more temperate climates contribute to the invasion of schistosome parasites into new areas such as Corsica (France) and Almeria (Spain) [3]. The genus *Schistosoma* contains six species that are of major pathological importance to humans: *Schistosoma haematobium*, *S. mansoni* , *S. japonicum* , *S. mekongi*, *S. intercalatum* and *S. guineensis* [4]. This study focuses on the species *S. mansoni* [2]. Freshwater snails serve as intermediate hosts for schistosomes and release cercariae, the parasite's infectious larval stage, into the environment. Upon contact, cercariae penetrate the host's skin and enter the vascular system where they develop into the adult stage [2]. Schistosomes are the only trematodes that have evolved separate sexes. Male and female *S. mansoni* worms mate, and as pairs they migrate into the mesenteric veins to produce eggs. These eggs either enter the intestinal lumen to be excreted into the environment, or they are flushed into other organs with the bloodstream. This way, many eggs reach organs such as the spleen and liver, where they lodge in sinusoids, enter the tissue and get enclosed in granulomas [2]. The eggs are the main trigger of organ damage, which motivated studies investigating the pathogenicity factors of eggs, such as IPSE or omega‐1 [5, 6]. In the liver, the eggs and surrounding granulomas induce a fibrotic remodelling, which ultimately leads to granulomatous liver cirrhosis with portal hypertension, splenomegaly, and formation of collateral venous circulation [7]. We have shown before that *S. mansoni* eggs activate the transcription factor c‐Jun in hepatocytes through IPSE, a well‐characterised compound of soluble egg antigens (SEA) [8]. c‐Jun acts as both a transcription factor and a proto‐oncogene in hepatic carcinogenesis [9]. c‐Jun can either dimerise as a homodimer or alternatively as a heterodimer with other members of the AP‐1 family to form the activator protein 1 (AP‐1) complex, capable of binding to AP‐1 promoter DNA sequences and regulating gene transcription [10]. In various scenarios of cellular stress such as ER stress associated with autophagy [11] and oxidative stress in acute hepatitis [12], c‐Jun also promotes hepatocyte survival. Indeed, exacerbated liver damage in hepatocyte‐specific c‐Jun knockout mice suggested a protective role of c‐Jun in the liver [13]. The aim of the current study was to evaluate the extent of an *S. mansoni* infection on murine liver damage under pharmacological inhibition of JNK/c‐Jun signalling and to investigate the underlying mechanisms.

## 2 Results

### 2.1 Binding of SP600125 to SmJNK Has No Effect on Parasite Fitness

**FIGURE 1:**
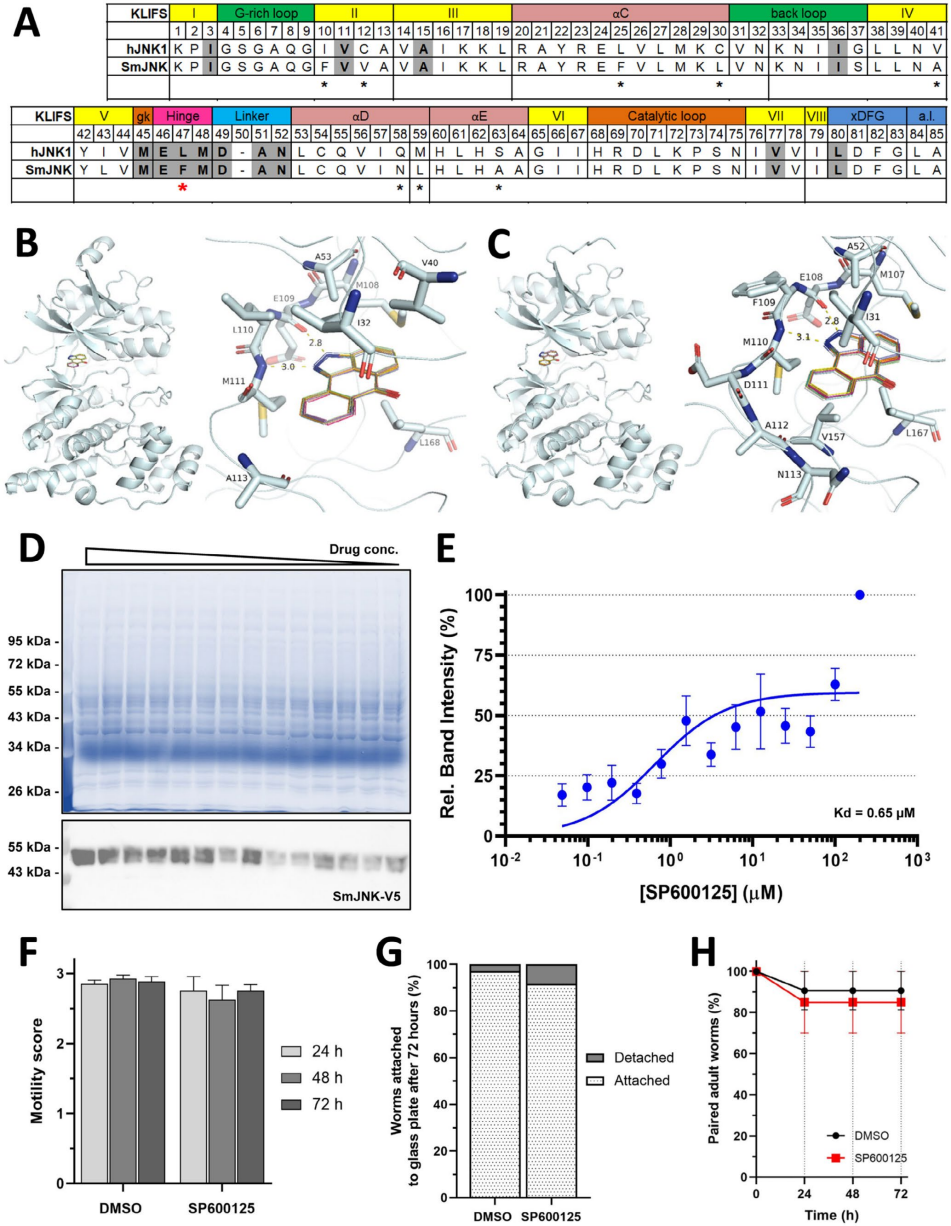
Binding of SP600125 to SmJNK showed no significant influence on *S. mansoni* motility, attachment and pairing. (A) Comparison of KLIFS residues within specified domains of hJNK1 and SmJNK protein sequences. Shaded residues indicate confirmed ligand‐target interactions between SP600125 and hJNK1. Asterisks indicate divergent residues. (B, C) Ribbon representation of hJNK1 (B) or SmJNK (C) with top 10 best scoring binding poses of SP600125. Zoomed region shows residues with side chains within 4 Å of distance to the ligand. Two hydrogen bonds are represented by dashed lines with distance in Å. (D, E) Isothermal dose–response fingerprint for intracellular SmJNK performed by cellular thermal‐shift assay. Quantification was performed by SDS‐PAGE/western blot (D) directed toward SmJNK using anti‐V5‐HRP conjugated antibody (1:5000). After quantification, the relative band intensities were plotted (E) as a function of ligand concentration to generate the fitted curve. SDS‐PAGE and western blot images are representative of the analysis of five independent experiments. Plotted data were given as average ± SEM, and the solid line represents the best fit of the data to the saturation binding curve model. (F–H) In vitro treatment of *S. mansoni* adult worms with 10 μM SP600125 for 3 days. 0.2% DMSO was used as control. Worms were scored for motility (F), attachment to the plate (G) and pairing status (H). In total, five worm couples per well (*n* = 3) were treated with the inhibitor or vehicle only (DMSO) for 3 days.

To specifically characterise the pharmacological impact of the JNK inhibitor SP600125 in *S. mansoni* ‐infected mice, we first assessed whether the inhibitor is able to bind to SmJNK. The kinase‐ligand interaction fingerprints and structures (KLIFS) for SP600125 are identical in mouse JNK1 and human JNK1 (hJNK1) as the according protein sequences are conserved among both species. Sequence analyses showed a difference of nine amino acids between the KLIFS residues (Figure 1A, asterisks) of hJNK1 and SmJNK, one of which is located in close proximity to the binding pocket (Figure 1A, Hinge 47, red asterisk). This difference may influence the binding affinity between the ligand and hJNK1 or SmJNK, respectively. In the crystal structure of hJNK1 bound to SP600125, two hydrogen bonds form [14]; one between the amino group of M111 and the nitrogen atom in position 1 of SP600125, and the second between the amide hydrogen group at the 2‐position of the inhibitor and the backbone carbonyl oxygen atom of E109. The bond length of the first is shorter than that of the corresponding hydrogen bond between M110 of SmJNK and the nitrogen atom in position 1 of the inhibitor (Figure 1B,C, 2.98 Å vs. 3.12 Å), suggesting a stronger binding affinity. In total, 11 residues of SmJNK are in close proximity (≤ 4 Å) to the ligand compared to nine residues in hJNK1. Moreover, the residue with direct contact to the ligand at Hinge 47 position has a bulky side chain group (F99) in SmJNK in comparison to the smaller side chain of L110 in hJKN1. Altogether, these results indicate that the pocket of SmJNK sterically influences the binding pose of SP600125 (Figure 1B,C). The consistency of the calculated conformations of hJNK1 and SmJNK with SP600125 was validated by calculation and correlation of DiffDock Confidence, SMINA Affinity, SMINA Minimised Affinity and SMINA Minimised RMSD according to protocols described previously (Figure S1) [15]. In addition, a cellular thermal shift assay (TSA) was performed to assess the binding of SP600125 to SmJNK. The apparent melting curve demonstrated that SP600125 has the ability to bind to the target, indicated by an increase in the thermal stability of the target protein (Figure S2). Further analysis by isothermal dose–response fingerprint for intracellular SmJNK showed that the inhibitor bound to SmJNK (Kd = 0.65 μM, Figure 1D,E), assuming comparability to the known inhibitory potential regarding hJNK1 (Ki = 0.19 μM, IC_50_ = 40 nM [16]). In line with the previous results, in vitro treatment of *S. mansoni* adult worms with SP600125 showed no effect on the parasite in regard to motility (Figure 1F) attachment (Figure 1G), and pairing status (Figure 1H). Altogether, the data suggested that SP600125 might bind SmJNK without visible effect on parasite fitness and viability during infection.

### 2.2 Systemic Inhibition of JNK Aggravated Liver Damage in *S. mansoni* Infection

**FIGURE 2:**
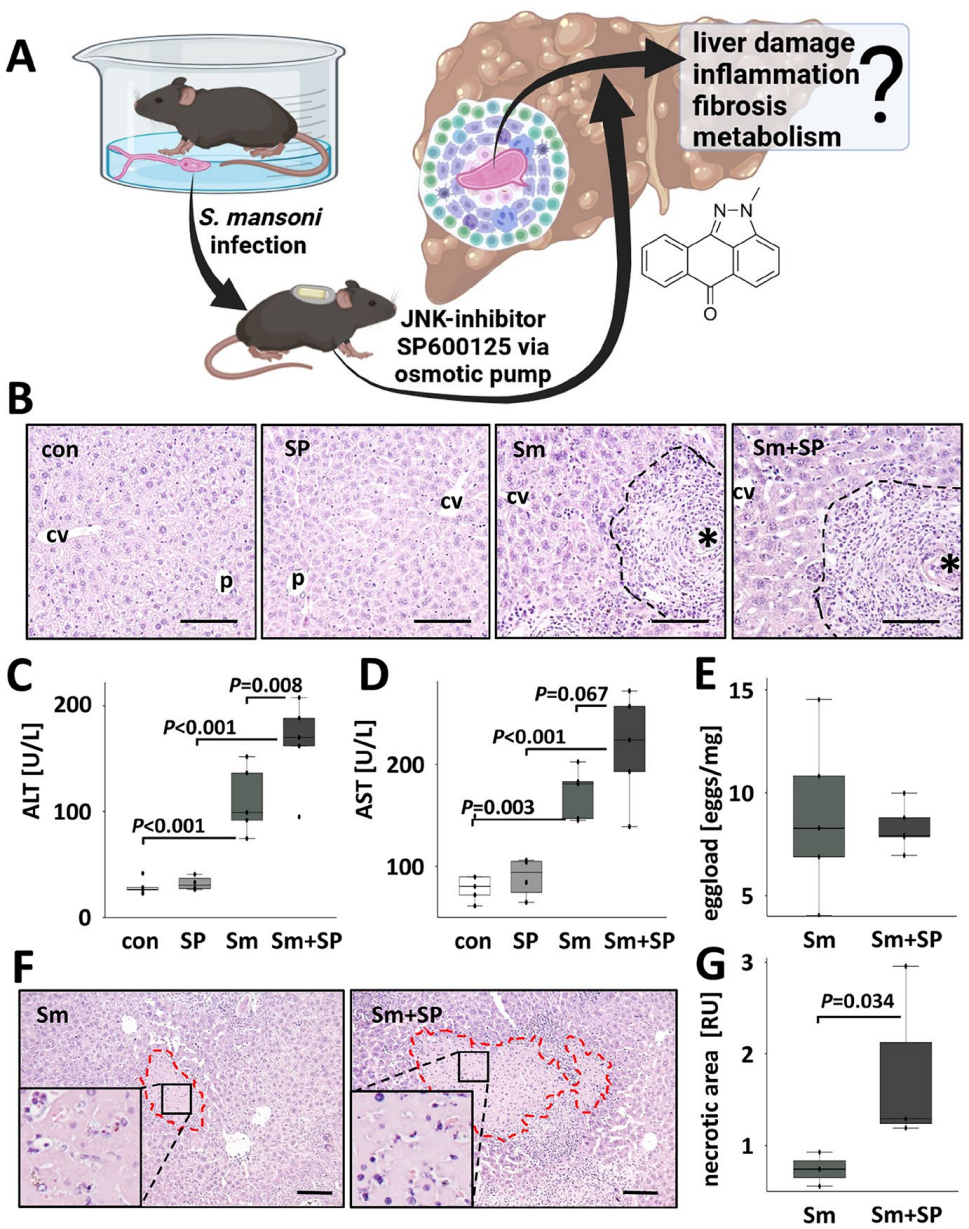
SP600125 exacerbates liver damage in *S. mansoni* ‐infected mice. (A) Schematic visualisation of the animal experiment and the addressed hypothesis, created with BioRender.com. (B) H&E staining visualised the granulomatous alterations in the liver of *S. mansoni* ‐infected mice. Black dashed line: Granuloma, *: *S. mansoni* egg, p: portal field, cv: central vein. Bars: 100 μm. Representative liver slices stained with H&E are shown. Enlarged pictures in Figure S4. Images of HE staining with a lower magnification to allow a more comprehensive visualisation of the hepatic tissue are shown in Figure S5. (C, D) JNK inhibitor (SP600125) treatment enhanced *S. mansoni* ‐induced serum ALT and AST levels. (E) Hepatic egg load was not affected by systemic JNK inhibition (Figure S6; visualisation of eggs in KOH‐digested liver). (F, G) Parenchymal necrosis was observed in three mice in each of the Sm and Sm + SP groups. Morphometric quantification of necrotic area in H&E‐stained tissue sections demonstrated expanded necrotic areas in *S. mansoni* ‐infected and SP600125‐treated mice. Bars: 100 μm. Representative liver slices stained with H&E are shown. Red dashed line: Border of necrotic area, the indicated area in the box was magnified in the lower left of each panel. Magnifications of microscopic images are shown in Figure S7. White bars: Uninfected control mice (con, *n* = 5; serum sampling failed in one case), light grey bars: SP600125‐treated mice (SP, *n* = 4) grey bars: *S. mansoni* ‐infected mice (Sm *n* = 5) and dark grey bars: *S. mansoni* ‐infected and SP600125 treated (Sm + SP, *n* = 5). The indicated *p*‐values were calculated by ANOVA and post hoc pairwise comparison of groups using Fisher's LSD or Levene's *t*‐test.

Eight‐week‐old C57BL/6J mice were infected with 100 *S. mansoni* cercariae of mixed sex (bisex, bs) as described before [13]. The JNK‐inhibitor SP600125 [16] or the carrier alone was continuously administered for the last 3 weeks of infection by a subcutaneously transplanted osmotic pump (Figure 2A; Figure S3).

None of the treated mice died as a result of the experimental procedures. *S. mansoni* eggs induced hepatic granulomas in all of the infected mice (Figure 2B; Figures S4 and S5). Hepatocellular damage resulted in increased serum ALT and AST levels (Figure 2C,D). Serum ALT levels were notably higher in *S. mansoni* ‐infected mice that were additionally treated with the JNK inhibitor SP600125 (Figure 2C). The hepatic egg load, measured by counting eggs in KOH digested liver tissue (Figure S6), was unchanged comparing *S. mansoni* ‐infected animals treated with SP600125 and untreated, infected animals (Figure 2E). Sm + SP‐treated mice exhibited more coagulative liver necroses in comparison to infected controls (Figure 2F,G; Figure S7). Interestingly, we identified TUNEL‐positive nuclei of hepatocytes in necrotic areas (Figure S8). While *S. mansoni* infection increased liver to body weight ratios as well as spleen to body weight ratios, both remained unaffected in the inhibitor group (Figure S9A,B) and showed a linear correlation among all animals of all experimental groups (Figure S9C). The transcriptional levels of hepatic caspases 1 and 3 were enhanced by *S. mansoni* infection, but they were not altered by additional SP600125 (Figure S9D,E). Since the integrity of hepatocytes is affected by c‐Jun‐controlled replication [13], we analysed the influence of SP600125 on the colocalisation of proliferating cell nuclear antigen (PCNA) with c‐Jun (Figure S10). Notably, c‐Jun/PCNA co‐staining of perigranulomatous hepatocellular nuclei in *S. mansoni* ‐infected mice appeared to be reversed by treatment with SP600125 (Black arrows, Figure S10).

### 2.3 SP600125 Treatment Enhanced Hepatic Inflammation in *S. mansoni* ‐Infected Mice

**FIGURE 3:**
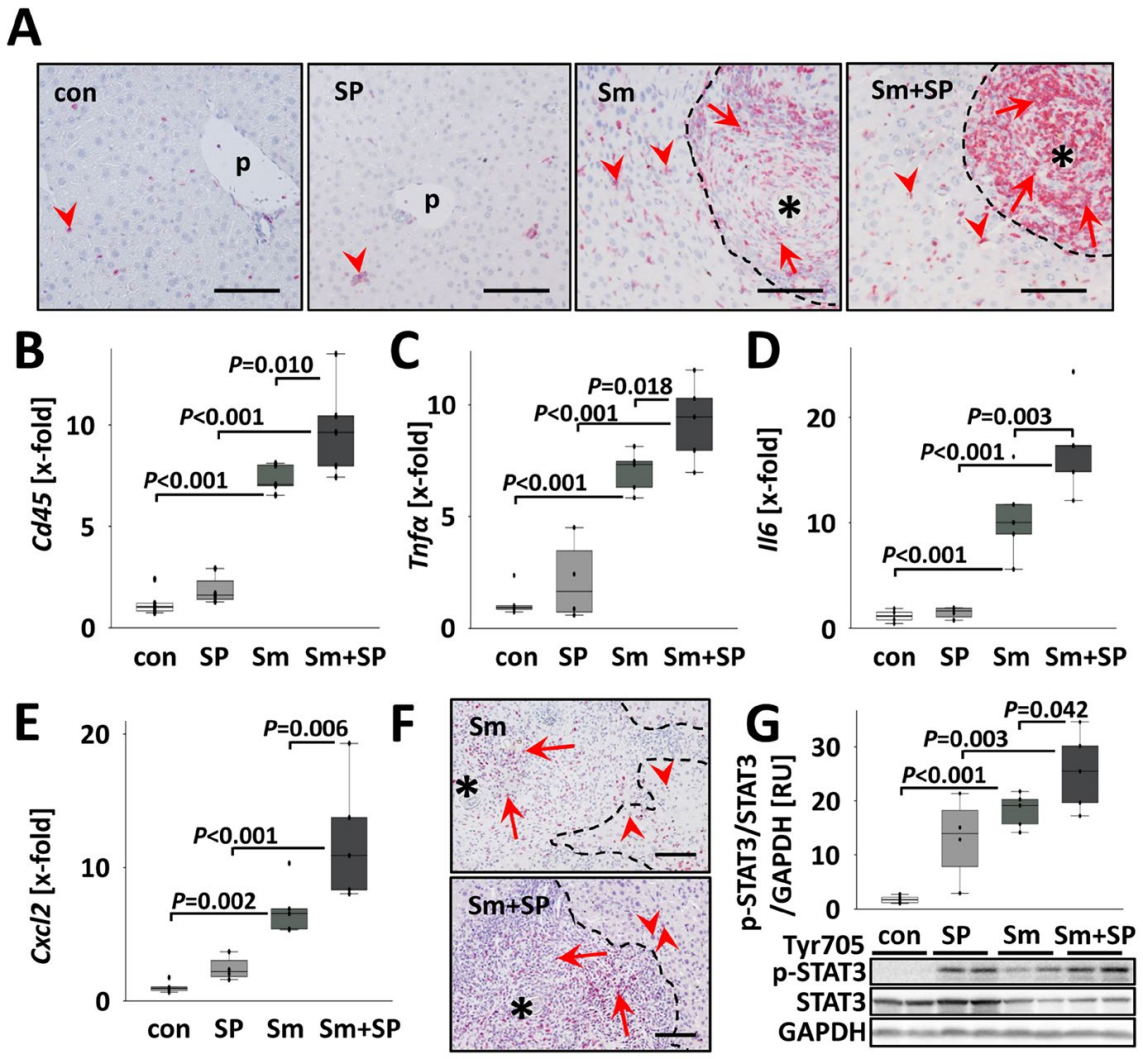
SP600125 increased hepatic inflammation in *S. mansoni* infected mice. (A) CD45 immunostaining visualised an immense hepatic infiltration of CD45^+^ leukocytes, especially in the granulomas (arrows) of inhibitor‐treated animals, but also into the parenchyma (arrowheads). p: portal field, *: *S. mansoni* egg. Bars: 100 μm. Magnifications of microscopic images are shown in Figure S11. (B) The quantification of hepatic *Cd45* expression revealed an additional increase of *Cd45* levels by SP600125 treatment in *S. mansoni* ‐infected mice. (C–E) The *S. mansoni* ‐induced expression of inflammatory cytokines *Tnfα*, *Il6* and *Cxcl2* was boosted by SP600125 treatment. (F) p‐STAT3 immunostaining revealed STAT3 activation inside the granulomas (arrows) but also in hepatocytes in direct vicinity (arrowheads). *: *S. mansoni* eggs, dashed line: Border of granulomas. Bars: 100 μm. Magnifications of microscopic images are shown in Figure S13. (G) Western blotting demonstrated a stronger hepatic activation of *S. mansoni* ‐induced STAT3 in SP600125‐treated mice. White bars: Uninfected control mice (con, *n* = 6), light grey bars: SP600125‐treated mice (SP, *n* = 4) grey bars: *S. mansoni* ‐infected mice (Sm, *n* = 5) and dark grey bars: *S. mansoni* ‐infected and SP600125 treated (Sm + SP, *n* = 5). The indicated *p*‐values were calculated by ANOVA and post hoc pairwise comparison of groups using Fisher's LSD.

CD45^+^ leukocytes infiltrated livers of *S. mansoni* ‐infected mice and constituted the majority of cells within the granulomas (Figure 3A; Figures S11 and S12).

**FIGURE 4:**
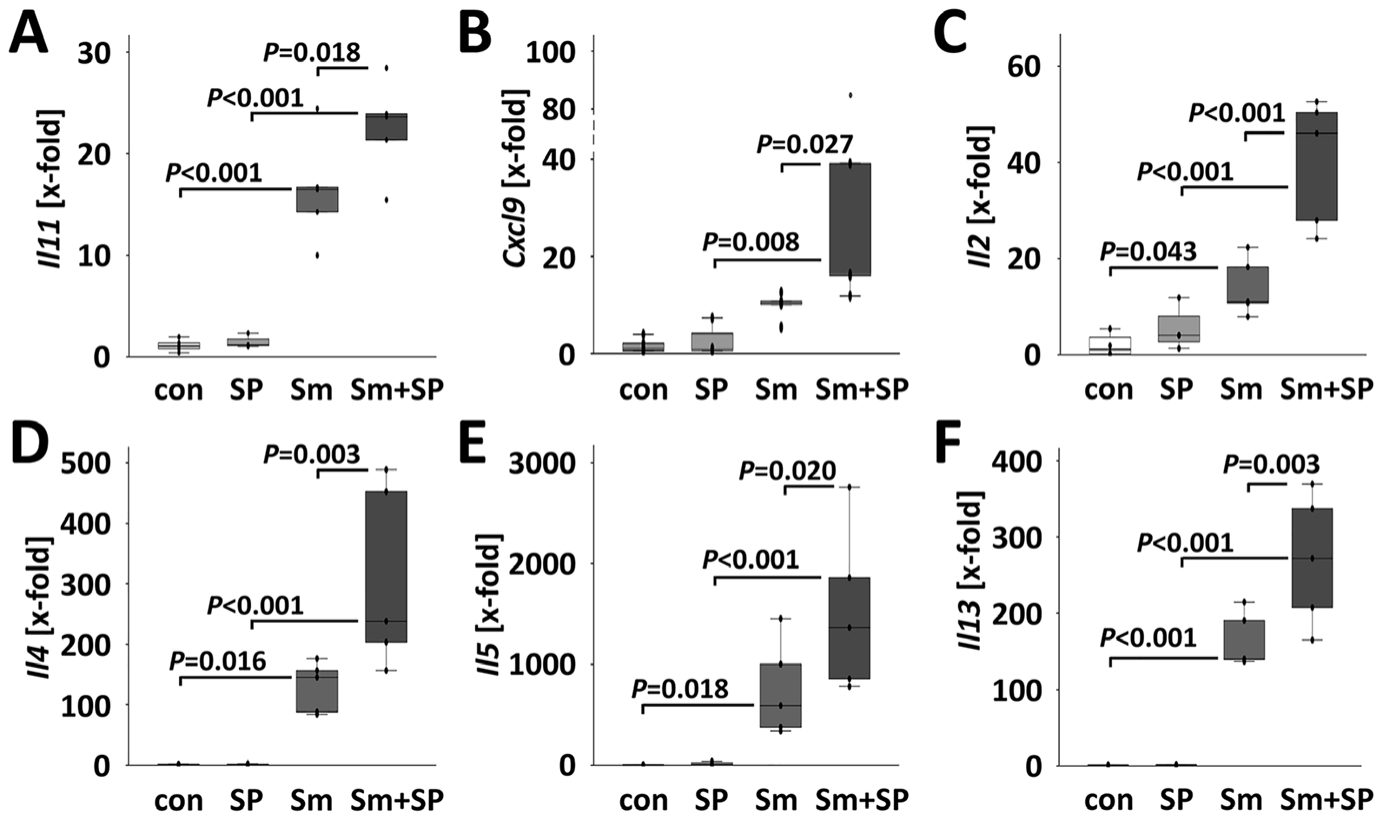
SP600125 increased hepatic cytokine expression in *S. mansoni* ‐infected mice. (A–F) Quantitative RT‐PCR demonstrated the additional increase of *S. mansoni* egg‐induced cytokines *Il11*, *Cxcl9*, *Il2*, *Il4*, *Il5* and *Il13* by SP600125. White bars: Uninfected control mice (con, *n* = 6), light grey bars: SP600125‐treated mice (SP, *n* = 4) grey bars: *S. mansoni* ‐infected mice (Sm, *n* = 5) and dark grey bars: *S. mansoni* ‐infected and SP600125 treated (Sm + SP, *n* = 5). The indicated *p*‐values were calculated by ANOVA and post hoc pairwise comparison of groups using Fisher's LSD.

The transcriptional levels of hepatic *Cd45* increased in infected mice treated with SP600125 (Figure 3B). In parallel, the increase in hepatic expression of *Tnfα*, *Il6* and *Cxcl2* (*Mip2*) was even more pronounced in Sm + SP treated mice (Figure 3C–E). Expression levels of the transcription factor STAT3 showed a similar pattern (Figure 3F,G; Figures S13 and S14). Additionally, other regulatory cytokines and chemokines like *Il11*, *Cxcl9*, *Il2*, *Il4*, *Il5* and *Il13*, which were induced by *S. mansoni* , showed even higher expression levels in infected mice upon JNK inhibition (Figure 4). In contrast, *Cxcl5* was not regulated, and the expression of *Il10* was induced by *S. mansoni* infection only (Figure S15) and remained unaffected by the inhibitor treatment. An overview of all cytokines that were analysed is presented in Figure S16.

### 2.4 JNK‐Inhibition Enhanced HSC Activation

**FIGURE 5:**
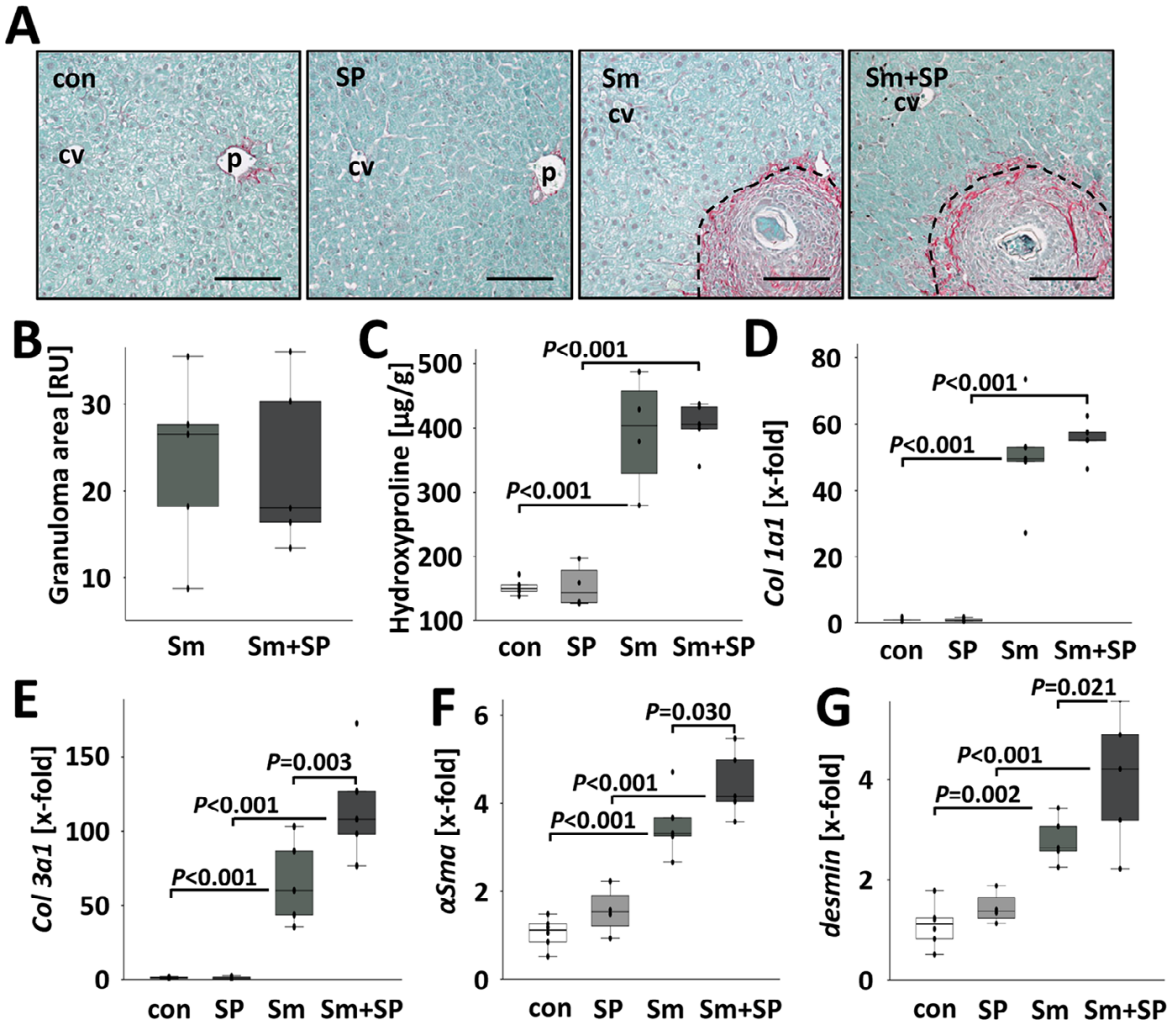
SP600125 promoted HSC transdifferentiation in *S. mansoni* ‐infected mice. (A) Sirius Red‐staining visualised the hepatic distribution of fibrillary collagens in red. Dashed line: Border of granulomas. Bars: 100 μm. Magnifications of microscopic images are shown in Figure S17. (B–D) Granuloma area, the increase of hepatic hydroxyproline, and hepatic Col1a1 expression were similar in both groups of *S. mansoni* ‐infected animals. (E–G) *S. mansoni* eggs‐induced expression levels of Col3a1, αSMA and Desmin further boosted by additional treatment with SP600125. White bars: Uninfected control mice (con, *n* = 6), light grey bars: SP600125‐treated mice (SP, *n* = 4) grey bars: *S. mansoni* ‐infected mice (Sm, *n* = 5) and dark grey bars: *S. mansoni* ‐infected and SP600125 treated (Sm + SP, *n* = 5). The indicated *p*‐values were calculated by ANOVA and post hoc pairwise comparison of groups using Fisher's LSD. *p* values are indicated.

As *Il11*, a master regulator of fibrosis [17], was strongly induced by *S. mansoni* infection and further boosted by inhibition of JNK (Figure 4A), we assessed the extent of hepatic fibrosis and the activation of hepatic stellate cells. Sirius red‐staining visualised the scale and pattern of hepatic fibrosis in *S. mansoni* ‐infected mice that were treated with SP600125 (Figure 5A; Figures S17 and S18). The major amount of red‐stained fibrillary collagens accumulated in the outer part of the granulomas. Morphometrically assessed sizes of Sirius red‐stained granuloma areas (Figure 5B), the quantification of hepatic hydroxyproline content (Figure 5C), and *Col1a1* expression indicated hepatic fibrogenesis in *S. mansoni* ‐infected animals (Figure 5D). Sm + SP‐treated mice showed similar and slightly more pronounced fibrogenesis. The hepatic levels of *Col3a1* as well as HSC activation markers *αSMA* and *desmin* were further elevated in SP600125‐treated mice that were infected with *S. mansoni* (Figure 5E–G).

### 2.5 SP600125 Promoted the Exhaustion of Hepatic Glycogen Stores in *S. mansoni* ‐Infected Mice

**FIGURE 6:**
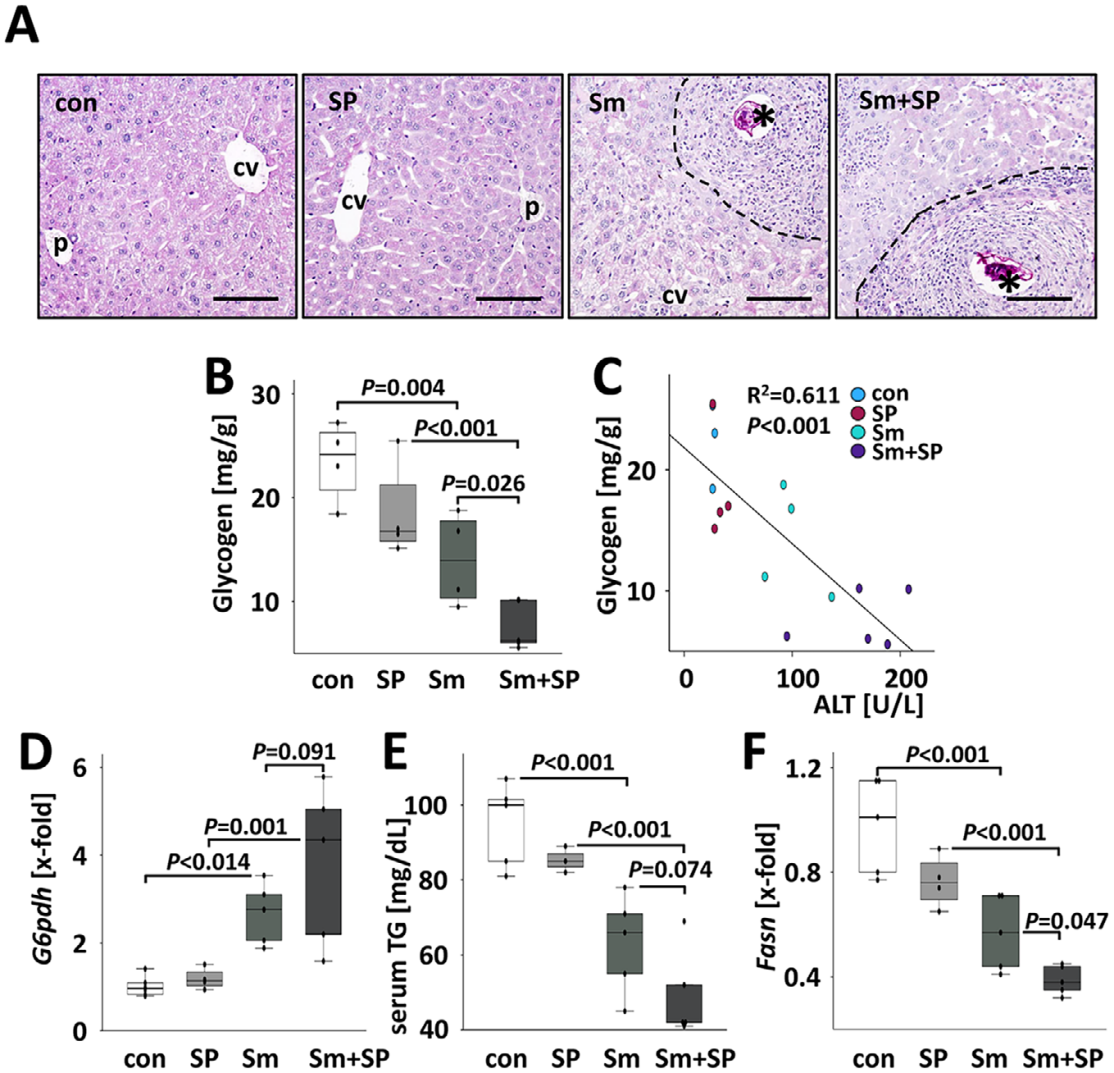
SP600125 exacerbated the exhaustion of hepatic glycogen stores in *S. mansoni* ‐infected mice. (A) PAS staining visualised glycogen exhaustion in the parenchyma and glycogen enrichment in the eggs of livers of *S. mansoni* ‐infected mice. Dashed line: Border of granuloma. Bars: 100 μm, *: Eggs. Magnifications of microscopic images are shown in Figure S19. (B) The quantity of glycogen in the liver was reduced in both groups of *S. mansoni* ‐infected mice, but most strongly in Sm + SP‐treated animals. Due to the material consuming analysis and its excellent reproducibility, glycogen quantification was performed once only with *n* = 4–5 representative samples of each group. (C) The amount of hepatic glycogen inversely correlated with the serum ALT levels. (D) Hepatic *G6pdh* expression was enhanced in *S. mansoni* ‐infected animals. (E) Serum triglyceride levels were reduced in *S. mansoni* ‐infected animals. (F) Hepatic expression of fatty acid synthase (*Fas*) was reduced in *S. mansoni* ‐infected animals. This effect was enhanced in mice additionally treated with SP600125. White bars: Uninfected control mice (con, *n* = 6), light grey bars: SP600125‐treated mice (SP, *n* = 4) grey bars: *S. mansoni* ‐infected mice (Sm, *n* = 5) and dark grey bars: *S. mansoni* ‐infected and SP600125 treated (Sm + SP, *n* = 5). The indicated *p*‐values were calculated by ANOVA and post hoc pairwise comparison of groups using Fisher's LSD.

We recently reported on the characteristic metabolic reprogramming of hepatocytes and the exhaustion of hepatic lipid and glycogen stores by schistosome eggs trapped in the liver [18]. Here, we analysed the influence of the pharmacological application of SP600125 on metabolically induced liver damage. Reduced glycogen stores in hepatocytes and a concurrent strong glycogen staining in *S. mansoni* eggs were visualised by PAS staining (Figure 6A; Figures S19 and S20). JNK inhibition on top of the *S. mansoni* infection further facilitated hepatic glycogen exploitation (Figure 6B). The amount of hepatic glycogen inversely correlated with serum ALT (Figure 6C).

The hepatic expression of glycolytic enzymes like *G6pdh* was induced by *S. mansoni* infection as published previously. The additive effect of SP600125 treatment was tendential, but not significant (Figure 6D). Glucose‐6‐phosphate dehydrogenase is a key player in cellular defence against oxidative stress. The additive effect of SP600125 treatment showed a trend, but was not statistically significant. Nevertheless, we further investigated the expression of catalase, an oxidative stress marker, that was previously shown to be strongly regulated in livers of *S. mansoni* infected hamsters [18]. *S. mansoni* ‐induced decrease in CAT expression tended to be stronger in *S. mansoni* ‐infected + SP600125‐treated mice (Figure S21). Notably, serum triglyceride levels (Figure 6E) were solely reduced by *S. mansoni* infection while the hepatic expression of rate‐limiting enzymes of lipid synthesis, such as *Fasn*, were further suppressed by additional JNK inhibition (Figure 6F).

## 3 Discussion

The current study demonstrated the exacerbation of liver damage by a systemic inhibition of JNK/c‐Jun signalling in *S. mansoni* ‐infected mice. Increased exhaustion of hepatic carbohydrate storage, a stronger inflammatory reaction, and likely reduced proliferation as well as enhanced activation of HSC contributed to the aggravation of liver injury. These results suggest a protective role of JNK/c‐Jun signalling in the mouse liver regarding hepatic inflammation, hepatic stellate cell activation and metabolic exhaustion upon *S. mansoni* infection.

We previously demonstrated that a hepatocyte‐specific knockout of c‐Jun resulted in enhanced liver damage [13]. The SP600125‐mediated (systemic JNK‐inhibition) increase in serum ALT in *S. mansoni* ‐infected mice appeared to be stronger than in *S. mansoni* ‐infected mice with only hepatocyte‐specific lack of c‐Jun. Our current data suggest that c‐Jun‐controlled hepatocyte proliferation is involved in SP600125‐mediated increase of hepatocellular damage (Figure S10). The increased inflammatory infiltration combined with an exceedingly enhanced cytokine expression is the most striking difference compared to a hepatocyte‐specific c‐Jun knockout and is the likely reason for the aggravation of liver damage and fibrosis in the current study. The hepatic induction of cytokine expression by *S. mansoni* is well characterised [19]. However, the boosted induction of cytokines by additional systemic JNK inhibition is a novel finding. Initially, SP600125 was described to inhibit the expression of inflammatory genes such as *COX‐2*, *IL‐2*, *IFN‐γ*, *TNF‐α*, and to prevent the activation and differentiation of primary human CD4 cell cultures [16]. Protective effects of SP600125 were observed for acetaminophen‐induced hepatic toxicity but not for carbon tetrachloride‐ or concanavalin A‐induced murine liver damage [20].

It has recently been reported that a fatal cytokine release syndrome (CRS) can be controlled by an aberrant STAT3 axis [21]. A STAT3‐linked, progressive, and fatal inflammatory syndrome in mice was characterised by elevated cytokine output and multiple organ dysfunctions that mimicked human CRS [21]. The enhanced activation of STAT3 in hepatic granulomas and the stronger induction of a broad range of cytokines in SP600125‐treated mice might be an analogous situation and a potential cause for enhanced liver damage. The induction of p‐Tyr STAT3 by inhibition of JNK with SP600125 has also been described in squamous carcinoma cells before [22]. STAT3 signalling has been considered an alternative pathway for kinases of the JAK and SRC families [23]. The latter are regulated by the receptor‐like protein tyrosine phosphatase CD45 [24] that was induced in the liver of *S. mansoni* infected mice and boosted by additional treatment with the JNK inhibitor SP600125 (Figure 2B).

The enhanced hepatic expression of IL‐2, IL‐5, IL‐13, or CXCL9 has been described in the context of schistosomiasis progression and generally in cirrhosis [25, 26, 27]. Therefore, it appears likely that the STAT3‐driven elevation of disease‐associated cytokines is the cause for the observed increase in liver damage. Understanding the immunomodulatory action of the parasite not only provides information on parasite–host interactions, but also forms the basis for the development of novel immunomodulatory strategies as new therapeutic approaches against *S. mansoni* . It is known that *S. mansoni* reprograms the host's immune response, creating a microenvironment that is favourable for the parasite [28]. *S. mansoni* uses various strategies to hijack the host's immune system, including dampening inflammatory cytokine responses and modulating Th1/Th2 cytokine profiles to evade the immune system and establish chronic infection [29]. Several studies provide evidence that Th2‐specific cytokine antagonism may be of general therapeutic benefit in preventing damaging tissue fibrosis resulting from Th2‐dominated inflammatory responses [27, 30, 31]. Apart from these preclinical studies, immunomodulation is not a primary therapy against *S. mansoni* yet. Nevertheless, understanding and modulating cytokine responses is crucial for understanding and potentially improving treatment strategies for hepatic schistosomiasis caused by *S. mansoni* , particularly in relation to the host's immune response toward the parasite.

Cytokines like IL‐11 or IL‐13 are known to be master regulators of fibrosis, especially in schistosomiasis‐associated fibrogenesis [17, 32]. Considering their functions and their boosted expression in SP600125‐treated *S. mansoni* ‐infected mice, it appears peculiar that the hepatic hydroxyproline levels were not significantly elevated. Nevertheless, *Col3a1* as well as HSC activation markers *desmin* and *αSma* were further induced by the inhibition of JNK in *S. mansoni* ‐infected mice. HSCs can amplify the hepatic inflammatory response, express growth factors that are critical for liver development, and are involved in the initiation and termination of liver regeneration [33]. Due to the critical role of HSCs in hepatic physiology and pathology, we hypothesise that the activation pattern, at least partially, contributed to the exacerbation of liver damage in SP600125‐treated *S. mansoni* ‐infected mice.

Energy derived from glycolysis may be critical for cell survival, and inhibition of glycolysis may result in hepatic cell death due to accumulation of end products or lack of glycogen [34]. In a rat model of cold ischaemia, glycogen depletion has been demonstrated as a pivotal factor for the progression of liver injury [34]. Moreover, the development of strategies to avoid hepatic glycogen depletion during cold preservation/reperfusion time is an emerging field in clinical science [35]. The depletion of hepatic glycogen stores in *S. mansoni* ‐infected mice was significantly enhanced by systemic JNK1/2 inhibition. Therefore, we also assume that the observed depletion of hepatic glycogen is a crucial factor contributing to fatal liver damage. Notably, JNK inhibition by SP600125 was also harmful in a model of hepatic I/R injury with similar effects as we observed, that is, increased hepatic leukocyte infiltration, enhanced expression of inflammatory cytokines, and aggravated serum ALT levels [36].

It is also noteworthy that the therapeutic properties of JNK inhibitor SP600125 have already been tested in mice infected with *S. japonicum* at a daily dose almost 40 times higher than in our experimental setting: Four weeks after abdominal infection with *S. japonicum* , each mouse was intraperitoneally injected with 50 mg/kg SP600125 once a day for 4 weeks [37]. In contrast to our results, the authors demonstrated that SP600125 reduced egg granuloma extent, fibrosis, and inflammation in the *S. japonicum* model [37], an effect that may be due to toxicity or decreased parasitic viability. The contrasting results of SP600125 treatment on hepatic schistosomiasis in the study by Song et al. [37] compared to our study are surprising at first glance. However, the treatment methods, *Schistosome* species, infection methods, and duration of infection are so different in both models that a direct comparison is not useful. Regarding the establishment of a therapeutic approach, inhibition of JNK/c‐Jun signalling does not appear to be effective, at least with regard to hepatic schistosomiasis in *S. mansoni* infection. On the contrary, our data show that JNK/c‐Jun signalling is important for hepatic integrity in *S. mansoni* infection. Therefore, the maintenance of hepatic JNK/c‐Jun signalling in *S. mansoni* infection should rather be considered as a therapeutic approach. The knockdown of SmJNK in schistosomula significantly increased the parasite's mortality, and the recovered adult worms showed considerable morphological alterations, especially in the tegument [38]. Taken into account that SP600125 is an inhibitor of human JNK with considerably reduced efficacy to schistosomal JNK, the immense concentrations in *S. japonicum* ‐infected mice might have harmed or killed the parasites, while the concentrations applied in this study only affected the host's JNK activity.

Finally, we presume that a boosted inflammatory response in combination with the activation of hepatic stellate cells and the almost complete hepatic glycogen depletion is the key factor for the exacerbation of liver damage in SP600125‐treated *S. mansoni* ‐infected mice. Moreover, the current results underline the protective role of JNK/c‐Jun‐signalling in the liver regarding hepatic inflammation, hepatic stellate cell activation, and metabolic exhaustion in *S. mansoni* infection. Our findings grant deeper insights into the processes induced in the liver upon egg deposition by *S. mansoni* . These results may stimulate further research to extend our understanding of this complex area, aiming to provide novel perspectives for therapeutic approaches in patients suffering from schistosomiasis.

## 4 Materials and Methods

### 4.1 Animal Model

*Biomphalaria glabrata* snails were used as intermediate hosts for maintaining the *S. mansoni* life cycle, and male C57BL/6JCrl mice were used as final hosts. At the age of 8 weeks, 12 randomly chosen mice were each treated with 100 cercariae of both sexes in a water bath at 30°C according to the recently published pre‐soaking procedure [39]. Six non‐infected mice were used as controls. At the age of 14 weeks, all mice were subcutaneously implanted with an osmotic pump (Alzet #1004) containing vehicle or vehicle and the JNK inhibitor SP600125 respectively corresponding to a previously published protocol [40]. Half of the animals from the *S. mansoni* ‐infected group as well as half of the uninfected mice received SP600125 or vehicle ([v/v]: 30% PEG400; 20% polypropylene glycol; 15% cremophor EL; 5% ethanol; 30% saline [0.9% NaCl w/v in ddH_2_O]) as a control via the osmotic pump. SP600125 (SP; 50 mg) was dissolved in 4 mL vehicle solution under sterile conditions. Each pump was back‐filled with 100 μL of the vehicle or vehicle + SP solution. The Alzet model 1004, with a delivery rate of 0.11 μL/h, provided a cumulative delivery of 33 μg of SP per mouse per day. After incubation for 48 h in sterile saline at 37°C, pumps were implanted subcutaneously at the top of the right foreleg by an experienced veterinarian (DZ). At the age of 17 weeks, mice were anaesthetised with isoflurane (4% v/v; Ecuphar GmbH), subsequently euthanised by cervical dislocation, and the organs were harvested for further analysis. Liver samples were shock‐frosted and stored at −80°C or preserved for histology as indicated below. Serum samples were stored at −80°C until analysis of aminotransferases (ALT, AST) and triglycerides (TG) by routine clinical chemistry on a Respons 910 Analyser using the appropriate kits according to the manufacturer's protocol (Diasys, Germany; ALT #127018010920, AST #126018010920, TG FS #157109911923). The success of *S. mansoni* infection was validated by evidence of eggs in stool and liver samples of the mice. Stool and liver of one animal in the Sm + SP group were free of parasitic eggs and the respective mouse showed no signs of infection. Therefore, this mouse was classified into the SP group. One mouse of the Sm group was identified as an extreme outlier indicating concomitant systemic diseases. In accordance with an experienced statistician (AW), this mouse was not considered for further analysis. The number of animals in the control group was increased to *n* = 6 by addition of three untreated mice that did not show a difference in any of the parameters analysed. By this procedure, four groups of mice were established: control (con, *n* = 6) uninfected + solvent; SP600125 (SP, *n* = 4) uninfected + JNK inhibitor; *S. mansoni* (Sm, *n* = 5) infected + solvent; *S. mansoni* and SP600125 (Sm + SP, *n* = 5) infected + JNK inhibitor. Abbreviations in brackets identify the groups throughout the paper.

The study is reported in accordance with ARRIVE guidelines [41]. All animal experiments have been done in accordance with the European Convention for the Protection of Vertebrate Animals used for experimental and other scientific purposes (ETS No 123; revised Appendix A) and were approved by the Regional Council Giessen (V 54‐19 c 20 15 h 01 GI 20/10 Nr. G 44/2019).

### 4.2 Molecular Docking, Binding Pose Analysis and Molecular Visualisations

The AlphaFold models of SmJNK (AF‐A0A3Q0KT26‐F1‐model_v4) and of the human JNK (AF‐P45983‐F1‐model_v4), as well as the SP600125 ligand, were used as input for the molecular docking analysis through Diffdock‐L [42]. The top 10 scoring binding poses were used for subsequent analysis. Interactions of SP600125 and hJNK1 were retrieved from the KLIFS database [43] and the respective structures (PDB ID #1UKI and #1PMV). Analysis of atom distances and structure visualisations were performed in pyMOL 2.5 [44].

### 4.3 Recombinant Expression of SmJNK

The full‐length sequence of *S. mansoni* JNK (Smp_172240.1) was subcloned into pMIB‐V5‐6xHis using *Kpn*I and *Not*I restriction sites, and the final vector was linearised with *Bgl*I endonuclease before transfection into Sf21 ( *Spodoptera frugiperda* ) insect cells for recombinant expression. Transporter 5 (Polysciences) was used as the transfecting reagent, and the transfection was performed according to the manufacturer's instructions. Recombinant cells were selected under the presence of 10 μg/mL of blasticidin (#ant‐bl‐1; InvivoGen, CA, USA). Cell culture was maintained in suspension in Sf900‐III medium at 27°C and shaking at 125 rpm. After selection, the presence of recombinant protein was confirmed by western blot using the whole cell lysate fraction.

### 4.4 Cellular Thermal Shift Assay for Intracellular SmJNK

Cellular thermal shift assay (CETSA) was performed to determine the apparent melting curve for intracellular SmJNK following the published protocol [45]. Briefly, a total of 30 million SmJNK‐expressing Sf21 cells per treatment group were used to establish the CETSA melting curve. The cells were cultured in the presence of 10 mM SP600125 or with DMSO (vehicle control) for 1 h before centrifugation and wash steps. The cell pellet was resuspended in 1 mL of PBS supplemented with protease inhibitor (SIGMA Fast Protease Inhibitor Cocktail [S8830; Sigma‐Aldrich]) and divided into 100 μL aliquots in 0.2 mL PCR tubes. Each tube was heated to a designated temperature ranging from 40°C to 67°C for 3 min followed by cooling for 3 min at room temperature. Cell lysates were prepared by centrifugation after two rounds of freeze–thaw cycles using liquid nitrogen. SDS‐PAGE and western blot analysis were performed to quantify the presence of soluble intracellular SmJNK. The data (relative band intensity) was plotted and fitted to a curve to obtain the apparent melting values using the Boltzmann Sigmoid equation. An isothermal dose–response fingerprint for intracellular SmJNK was performed similarly to the protocol described above, except that the concentration of SP600125 varied instead of the temperature during the heating step. Data were plotted and fitted to a saturation binding curve function (rectangular hyperbola, binding isotherm) within GraphPad Prism version 8.0.2 (GraphPad Software, Boston, MA, USA, www.graphpad.com).

### 4.5 Treatment of Adult *S. mansoni* Worms With SP600125

Freshwater snails ( *Biomphalaria glabrata* ) served as the intermediate hosts for the ST (Liberia) strain of *S. mansoni* [46]. As final hosts, golden hamsters ( *Mesocricetus auratus* , Janvier, France) at the age of 8 weeks were infected by the paddling method using up to 1750 cercariae per hamster [39]. Worms were collected by hepatoportal perfusion 46 days post‐infection (p.i.). For regular maintenance in vitro, worms were cultured in M199 medium (Merck, Germany) supplemented with 10% Newborn Calf Serum (NCS; Merck), 25‐mM HEPES (ThermoFisher Scientific, USA), and 1% ABAM solution (100 U/mL penicillin, 100 μg/mL streptomycin and 250 μg/mL amphotericin B; Merck) at 37°C in a 5% CO_2_ atmosphere [47].

One day after perfusion, five couples per well (*n* = 3) were transferred to 6‐well culture plates in 2.5 mL of supplemented medium. SP600125 was added to a final concentration of 20 μM and 0.4% DMSO was used as control. The medium was changed every day, followed by the addition of compound or DMSO. Worms were analysed under an inverted microscope attached to a camera (Zeiss Primovert—Axiocam 208 colour, Oberkochen, Germany). The scoring for motility, attachment and pairing status was performed as previously described [48]. Three independent biological replicates were performed. Two‐way ANOVA with uncorrected Fisher's LSD test was used to analyse data regarding adult worm motility, attachment and pairing.

### 4.6 Western Blot Analysis

Protein analysis was performed according to the previously published protocols [8]. The following primary antibodies were used (1:1000 dilution in 5% BSA): p‐STAT3 (Cell Signalling; #9145), STAT3 (Cell Signalling; #4904S), GAPDH (Proteintech; #60004‐1‐Ig). *S. mansoni* JNK was detected with 1:5000 V5 tag monoclonal antibody, HRP conjugated (Invitrogen; #R961‐25) in 5% dry milk. Secondary antibodies used (1:5000 in 5% dry milk) were: Goat anti‐rabbit IgG (Cell Signalling; #7074), horse anti‐mouse IgG (Cell Signalling; #7076).

### 4.7 Immunohistochemistry and Sirius‐Red Staining

Liver sections of 3 μm, fixed in formalin and embedded in paraffin, were used for immunohistochemistry (IHC) with the ImmPRESS AP REAGENT KIT (Vector Laboratories Inc., USA; #MP‐5401). Paraffin removal, unmasking and blocking protocols were published previously [49]. Primary antibodies used were: CD45 (Cell Signalling; #70257), p‐STAT3 (Cell Signalling; #9145). Colour reaction was developed with the Permanent AP Red Kit (Zytomed, Germany; #ZYT‐ZUC001‐125). Sirius‐red staining was performed as described in López‐De León and Rojkind [50].

### 4.8 Morphometric Analysis

Microphotographs (50× magnification) of three randomly chosen areas of Sirius‐red and H&E‐stained liver slices were analysed via the ImageJ software. Necrotic areas in H&E‐ or fibrotic areas in Sirius‐red stained slices were manually selected and replaced by a blue label area (BLA, hue δ147/184, saturation 155/255, brightness 117/255), whereas areas representing major vessels or fissures of the tissue were labelled in yellow (YLA, hue 38/52, saturation 0/255, brightness 255/255). Afterwards, the size of the fibrotic or necrotic area was compared to the total tissue area (TTA).

$$\frac{\mathrm{BLA}}{\text{total image area}-\mathrm{YLA}}\;\to \;\frac{\mathrm{BLA}}{\mathrm{TTA}}\;\to \;\frac{\text{fibrotic area}}{\mathrm{TTA}}$$

### 4.9 Quantitative Real‐Time PCR

Messenger‐RNA (mRNA) was isolated using the RNeasy Mini Kit (Qiagen, Germany; #74106) and synthesised using the iScript cDNA Synthesis Kit (Bio‐Rad, USA; #1708891) according to the manufacturer's protocols. RT‐qPCR was performed adhering to published protocols [51] and analysed using the 2^−ΔΔCT^ method [52]. Primer sequences are presented in the Table S1.

### 4.10 Hydroxyproline Assay

Hydroxyproline was quantified as previously described [53].

### 4.11 Quantification of *S. mansoni* Eggs

Liver tissue of infected mice was digested in a 5% potassium hydroxide (KOH) solution for 16 h at 37°C. *S. mansoni* eggs were counted under 400× magnification and the number of eggs per milligram of tissue was assessed.

### 4.12 Statistical Analysis

Statistical analysis was performed with SPSS 26.0. (SPSS Inc., IBM Corp., Armonk, NY). In regard to the exploratory nature of the current study, one‐way ANOVA with post hoc Fisher's least significant differences (LSD) as well as *t*‐tests were used to calculate pairwise differences between animal groups [54]. The coefficient of determination *R* ^2^ with the respective *p*‐value was determined using linear modelling. Relevant *p*‐values are indicated in the corresponding figures. The upper hinge of the box represents the 75th percentile; the lower hinge represents the 25th percentile. The line in the box indicates the median value of the data. The ends of the vertical lines represent the minimum and maximum.

## Author Contributions

E.R. and M.R. conceived the project and directed the study. F.S., L.K., E.R. and M.R. wrote the manuscript. F.S., L.K., M.H., V.B., H.M., A.T., D.Z., S.H., M.F.M., B.P.M., C.L. and A.S. performed experiments. F.S., L.K., M.H., V.B., D.Z., S.H., A.W., M.B.‐R., D.G., B.P.M., A.S., F.H.F., C.G.G., E.R. and M.R. analysed, interpreted and discussed the data. D.Z., S.H., M.F.M., M.B.‐R., D.G., F.H.F. and C.G.G. contributed samples and materials. L.K., M.R. and A.W. were involved in statistical analysis. All authors reviewed the manuscript.

## Conflicts of Interest

The authors declare no conflicts of interest.

## Supporting information


**Data S1:** liv70260‐sup‐0001‐DataS1.zip.

## Data Availability

The data that support the findings of this study are available on request from the corresponding author.
