# Supplementary material for: Pharmacological Inhibition of c‐Jun N‐Terminal Kinase Activity Exacerbates Liver Damage in Schistosoma mansoni Infected Mice
Source: Liver Int. 2025 Aug 2;45(9):e70260. doi: 10.1111/liv.70260 (PMC12317361; doi:10.1111/liv.70260)
Supplement: Supplementary file 1 — Data S1: liv70260‐sup‐0001‐DataS1.zip. [file LIV-45-0-s001.zip › Supplementary Table CAPTION.docx]

Supplementary Table 1: Primer sequences used for qRT-PCR analyses
