# Supplementary material for: Pharmacological Inhibition of c‐Jun N‐Terminal Kinase Activity Exacerbates Liver Damage in Schistosoma mansoni Infected Mice
Source: Liver Int. 2025 Aug 2;45(9):e70260. doi: 10.1111/liv.70260 (PMC12317361; doi:10.1111/liv.70260)
Supplement: Supplementary file 1 — Data S1: liv70260‐sup‐0001‐DataS1.zip. [file LIV-45-0-s001.zip › liv70260-sup-0001-supinfo.pdf]

SFig. 1

A

Prediction	DiffDock Confidence	SMINA Affinity
rank1_confidence0.30.sdf	0.3	-6.7333
rank2_confidence0.27.sdf	0.27	-6.09067
rank3_confidence0.27.sdf	0.27	-6.8032
rank4_confidence0.26.sdf	0.26	-6.66281
rank5_confidence0.26.sdf	0.26	-6.38989

B

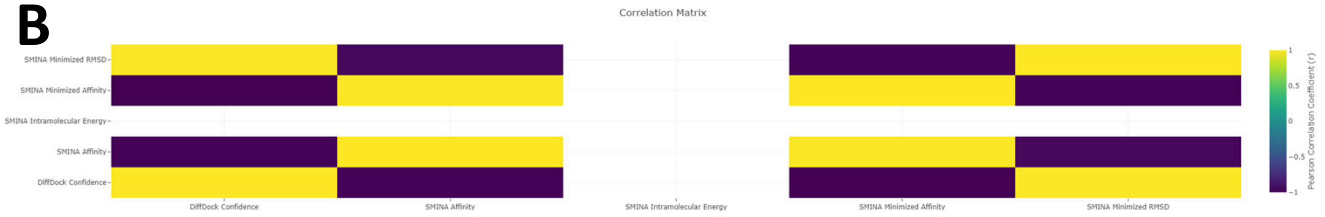

C

Prediction	DiffDock Confidence	SMINA Affinity
rank1_confidence0.09.sdf	0.09	-6.47931
rank2_confidence0.05.sdf	0.05	-4.51422
rank3_confidence0.05.sdf	0.05	-5.61
rank4_confidence0.03.sdf	0.03	-6.13783
rank5_confidence-0.02.sdf	-0.02	-5.6455

D

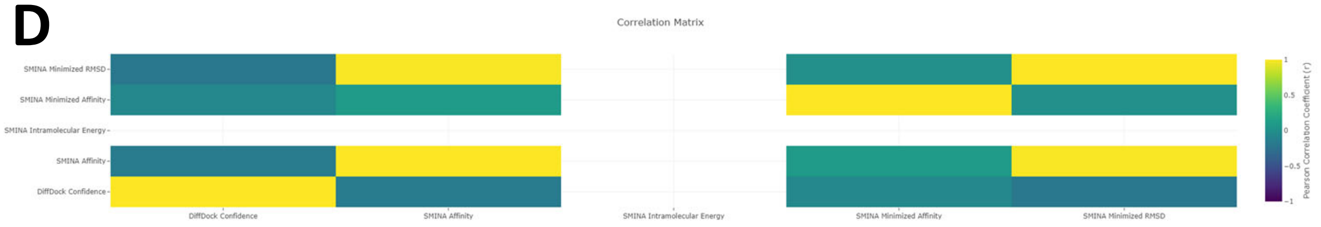

SFig. 1: Validation of conformational consistency. The consistency of the calculated conformations of hJNK1 and SmJNK with SP600125 was validated by calculation and correlation of DiffDock Confidence, SMINA Affinity, SMINA Minimized Affinity, and SMINA Minimized RMSD according to protocols described before [doi.org/10.48550/arXiv.2210.01776, doi.org/10.1021/acs.jcim.1c00203, doi.org/10.1002/jcc.21334]. DiffDock and SMINA agree with one-another's predictions which fundamentally means that the two models betting on the same ligands. **(A and B)** hJNK1 and **(C and D)** Sm JNK.

SFig. 2

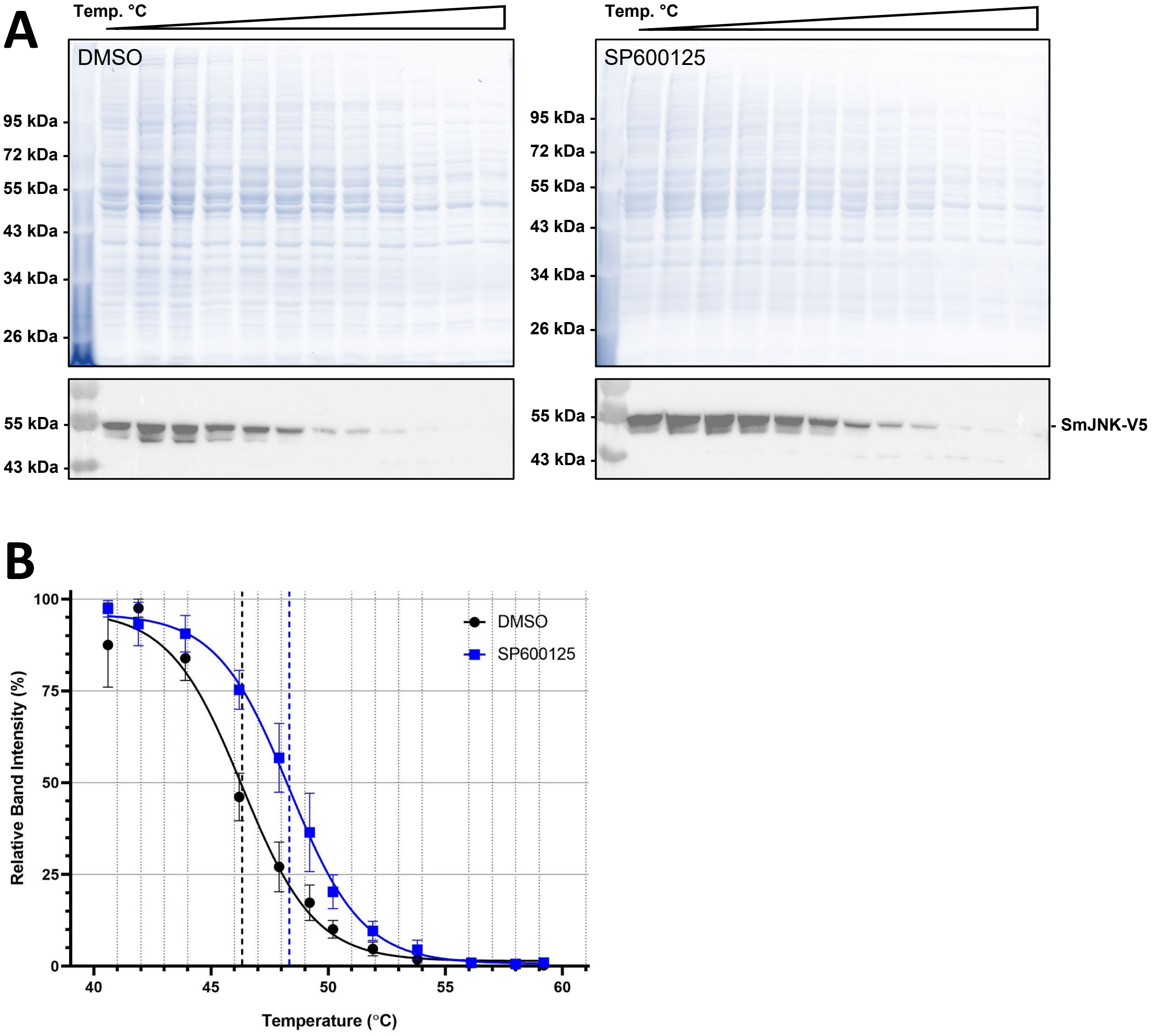

SFig. 2: Determination of the apparent melting curve for intracellular SmJNK performed by cellular thermal-shift assay. (A) The soluble fraction of whole cell lysates were analyzed by SDS-PAGE after heating treatment from 40 to 67 °C in the presence (SP600125) or absence (DMSO) of compound for 1 hour in cell culture. Quantification was performed by western blot directed toward SmJNK using anti-V5-HRP conjugated antibody (1:5000). SDS-PAGE and western blot images are representative of the analysis of three independent experiments. **(B)** After quantification the relative intensities of bands indicating soluble thermostable SmJNK were plotted to generate a fitted curve. Plotted data were given as average ± s.e.m (n=3) and the solid line represent the best fit of the data to the Boltzmann sigmoid equation within GraphPad Prism v8.0.

SFig. 3

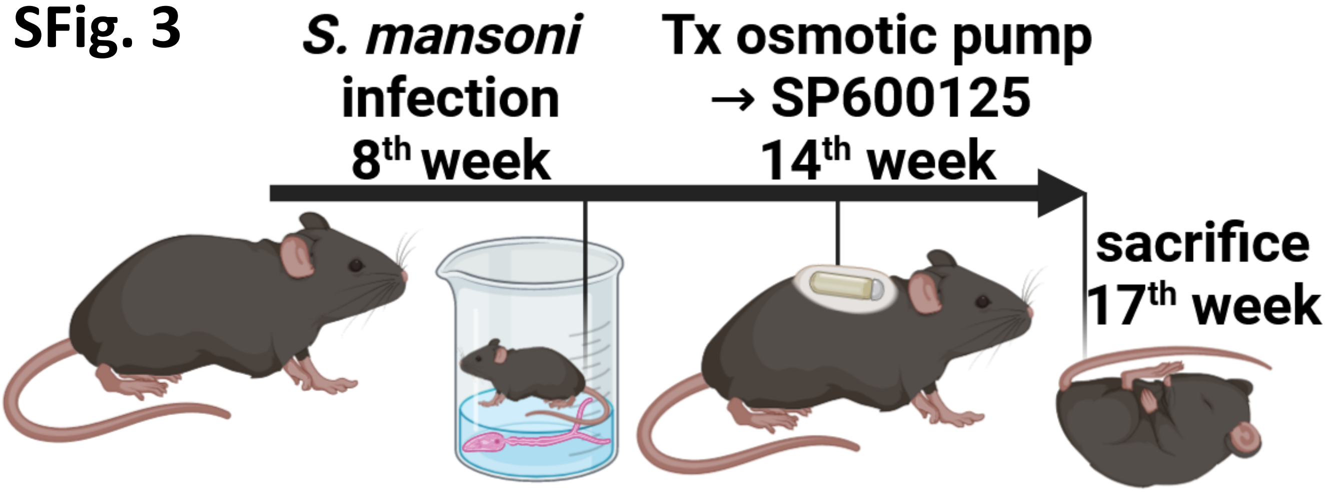

SFig. 3: Schematic presentation of the experimental setting. Mice of the *Sm* and *Sm*/SP groups were infected with cercariae of the parasite *S. mansoni* at the age of 8 weeks. Controls were treated in the same way without cercariae. An osmotic pump was transplanted subcutaneously at the age of 14 weeks to administer the JNK Inhibitor SP600125 or the carrier solution for control for 3 weeks.

SFig. 4

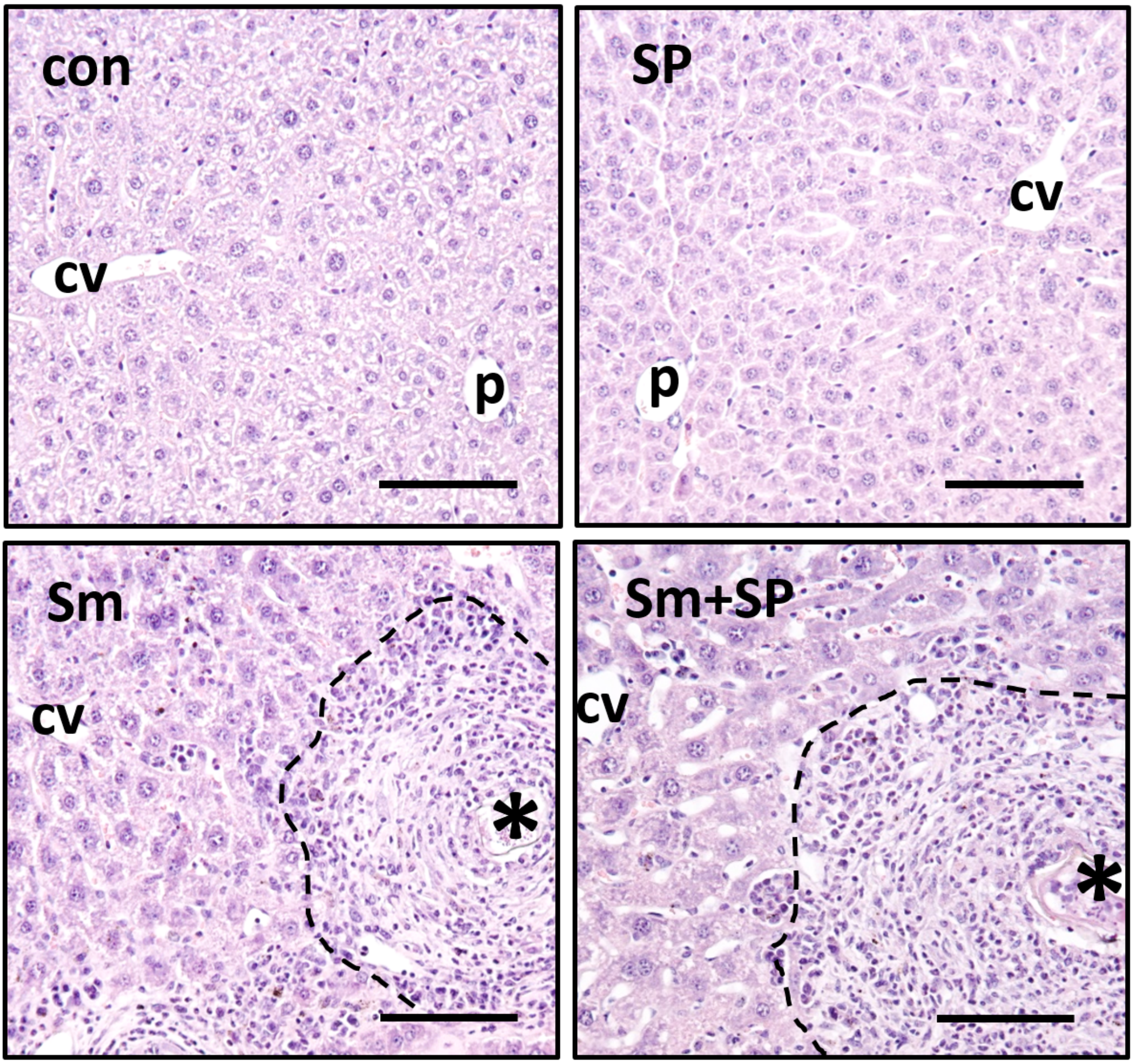

SFig. 4: Enlarged Figure 2B. H&E staining visualized the granulomatous alterations in the liver of *S. mansoni*-infected mice. Black dashed line: granuloma, * *S. mansoni* egg, p: portal field, cv: central vein, bars: 100µm. Representative liver slices stained with H&E are shown.

SFig. 5

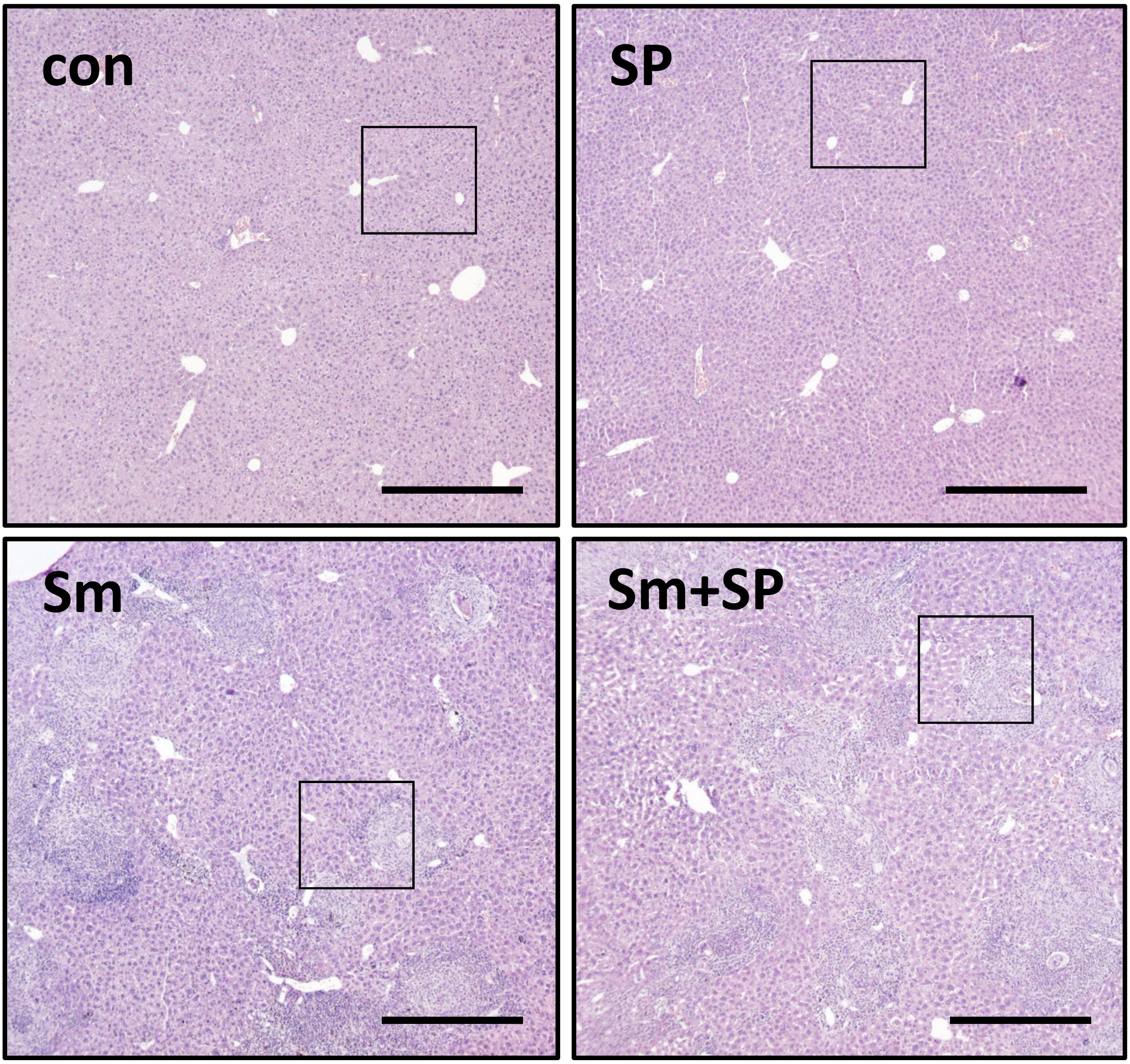

SFig. 5: Images of HE stainings with 50x magnification. Boxes indicate areas shown in figure 2B. H&E staining visualized the granulomatous alterations in the liver of *S. mansoni*-infected mice. Bars: 500µm. Representative liver slices stained with H&E are shown.

SFig. 6

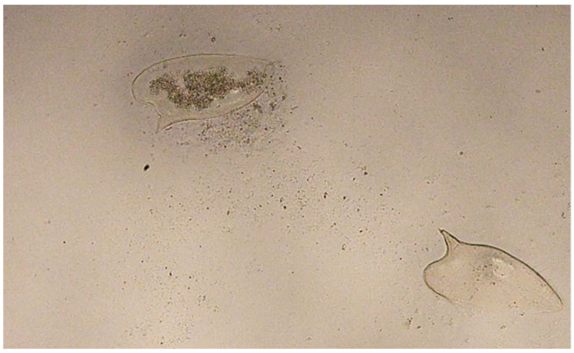

SFig. 6: Visualization of eggs in KOH-digested liver.

SFig. 7

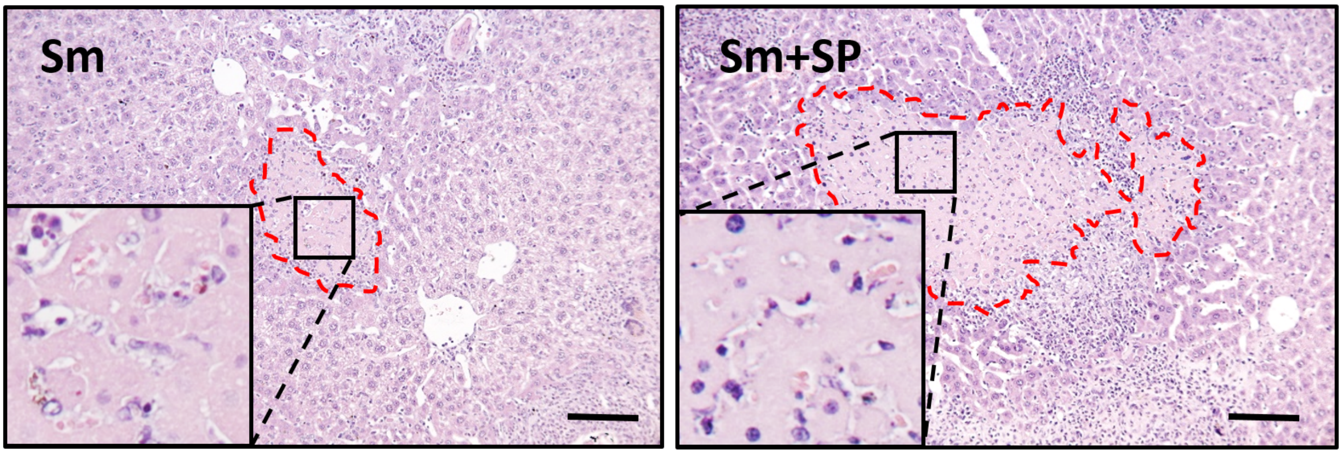

SFig. 7: Enlarged Figure 2F. Parenchymal necrosis was observed in three mice in each of the Sm and Sm+SP groups. Morphometric quantification of necrotic area in H&E stained tissue sections demonstrated expanded necrotic areas in *S. mansoni*-infected and SP600125-treated mice, bars: 100µm. Representative liver slices stained with H&E are shown. Red dashed line: border of necrotic area, the indicated area in the box was magnified in the lower left of each panel.

SFig. 8

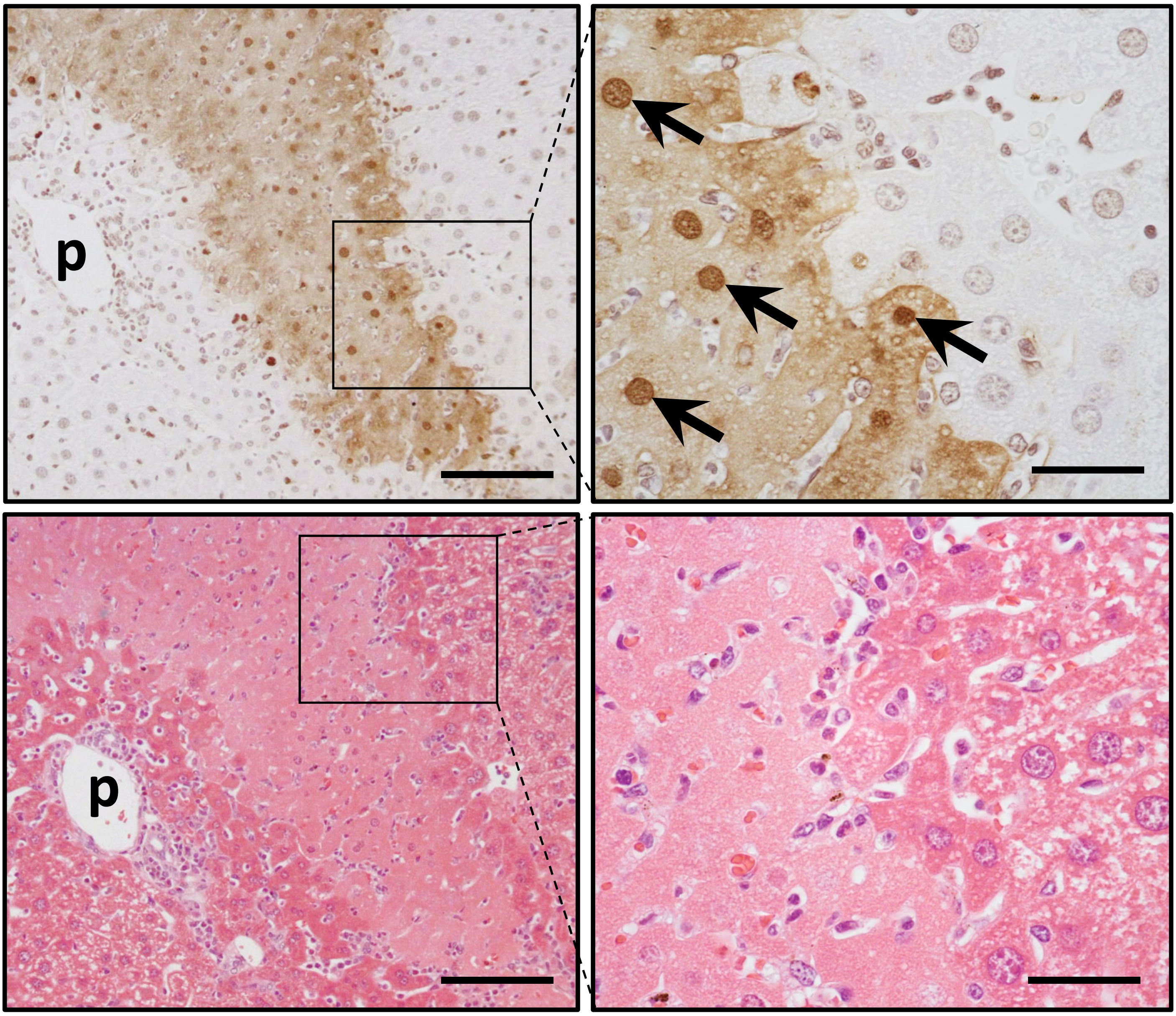

SFig. 8: TUNEL assay (upper panels) and H&E staining (lower panels) of a necrotic area on consecutive liver slices. Arrows indicate TUNEL-positive nuclei of hepatocytes in necrotic areas. Boxes indicate magnified areas shown in panels on the right. Magnification 200x (left panels) 1000x (right panels), bars: 100µm (left) and 20µm (right). Representative stainings are shown.

SFig. 9

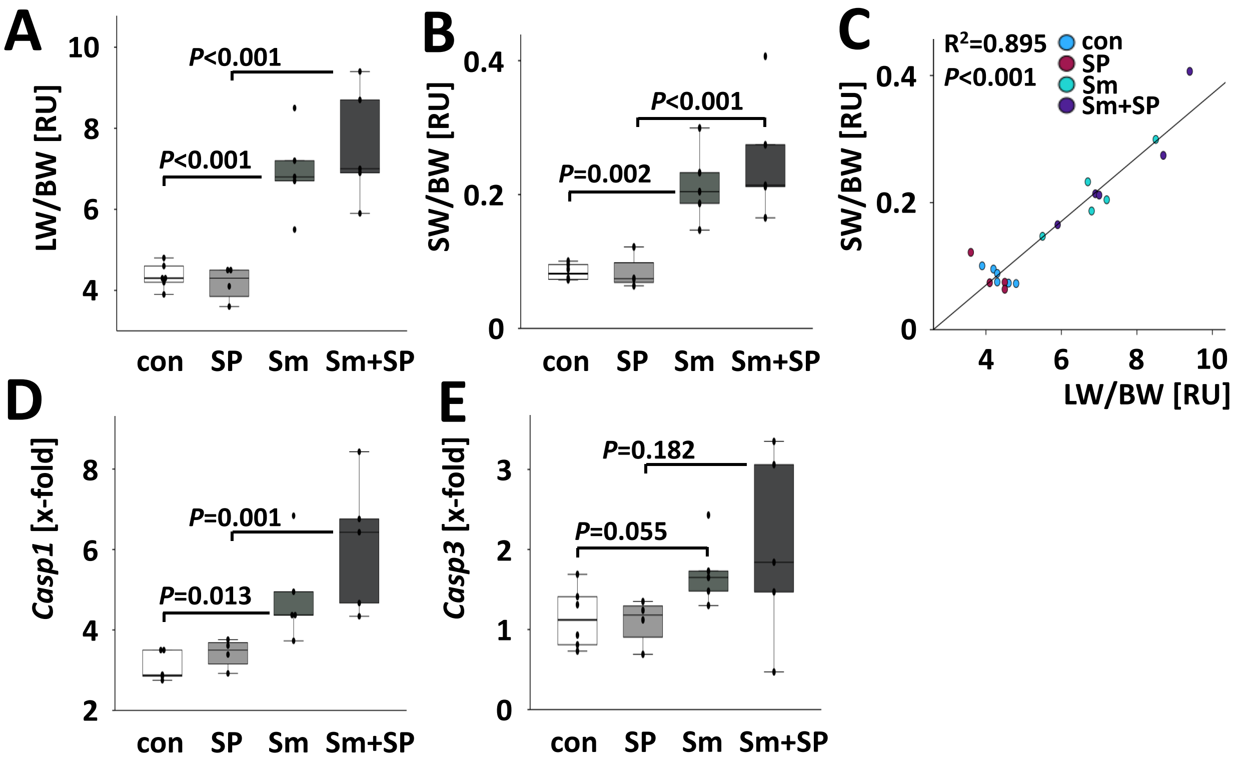

SFig. 9: Organ weight and caspase expression. (A) Liver weight (LW) to body weight (BW) ratio. **(B)** Spleen weight (SW) to body weight (BW) ratio. **(C)** Dot blot and linear correlation analysis SW/BW to LW/BW. **(D-E)** Hepatic *Casp1* and *Casp3* expression levels. White bars: uninfected control mice (con n=5; serum sampling failed in one case), light grey bars: SP600125-treated mice (SP n=4) grey bars: S. mansoni-infected mice (Sm n=5) and dark grey bars: S. mansoni-infected and SP600125 treated (Sm+SP n=5). Each qRT-PCR was repeated at least 3-times.

SFig. 10

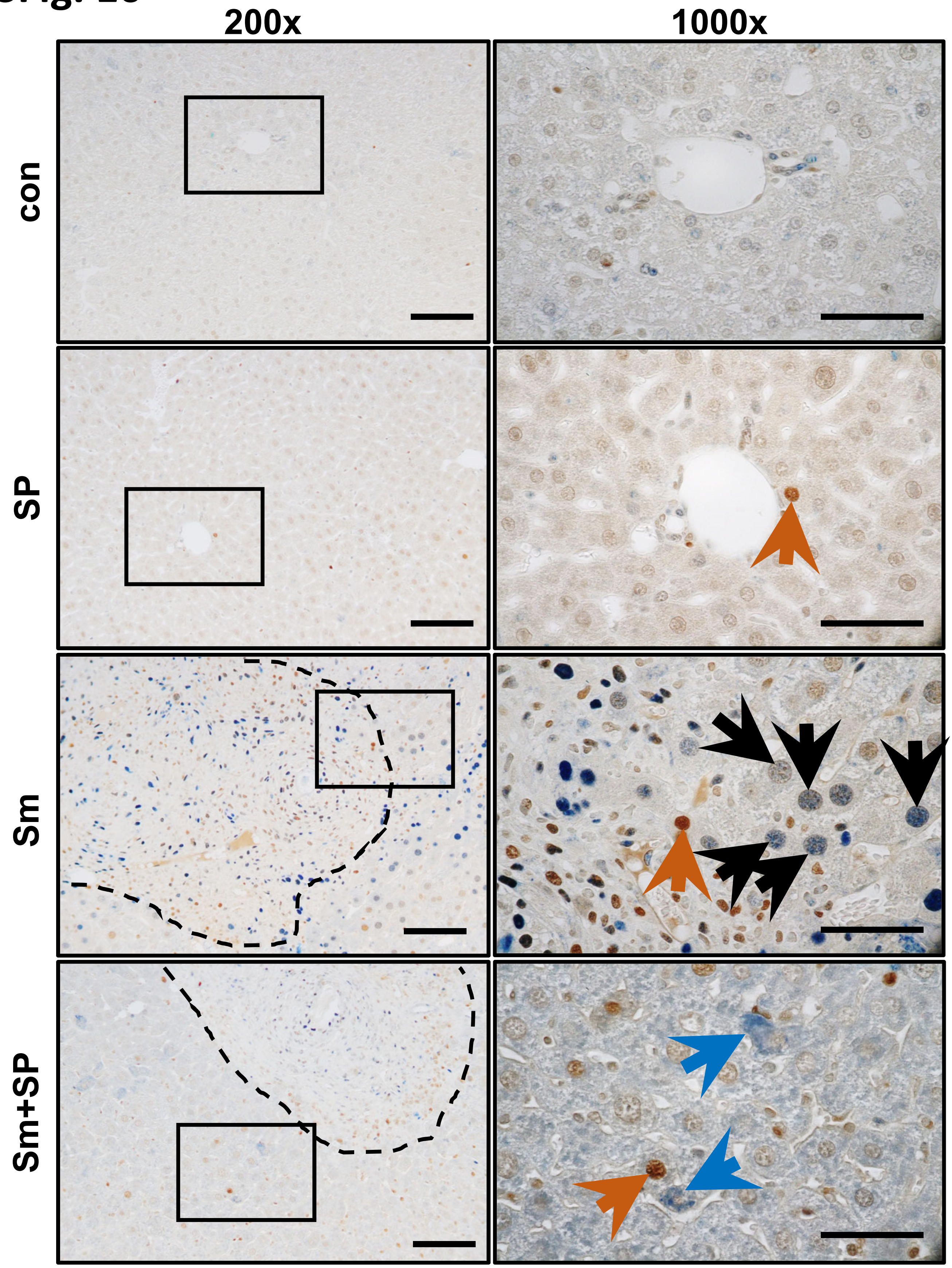

SFig. 10: Hepatic co-expression of c-Jun and Proliferating Cell Nuclear Antigen (PCNA) in mice infected with *S. mansoni* is reversed by JNK inhibition. Co-immunostainings of c-Jun (blue) and PCNA (brown) of the four treatment groups are shown. Black arrows indicate c-Jun/PCNA co-immunostaining of hepatocyte nuclei in *S. mansoni*-infected mice. Brown arrows indicate hepatocyte nuclei that are stained for PCNA. Blue arrows indicate cytoplasmic c-Jun in hepatocytes of mice that were infected with *S. mansoni* and treated with SP600125. Magnification 200x left, 1000x right panels; bars 100µm left and 50µm right. Representative microphotographs are depicted.

SFig. 11

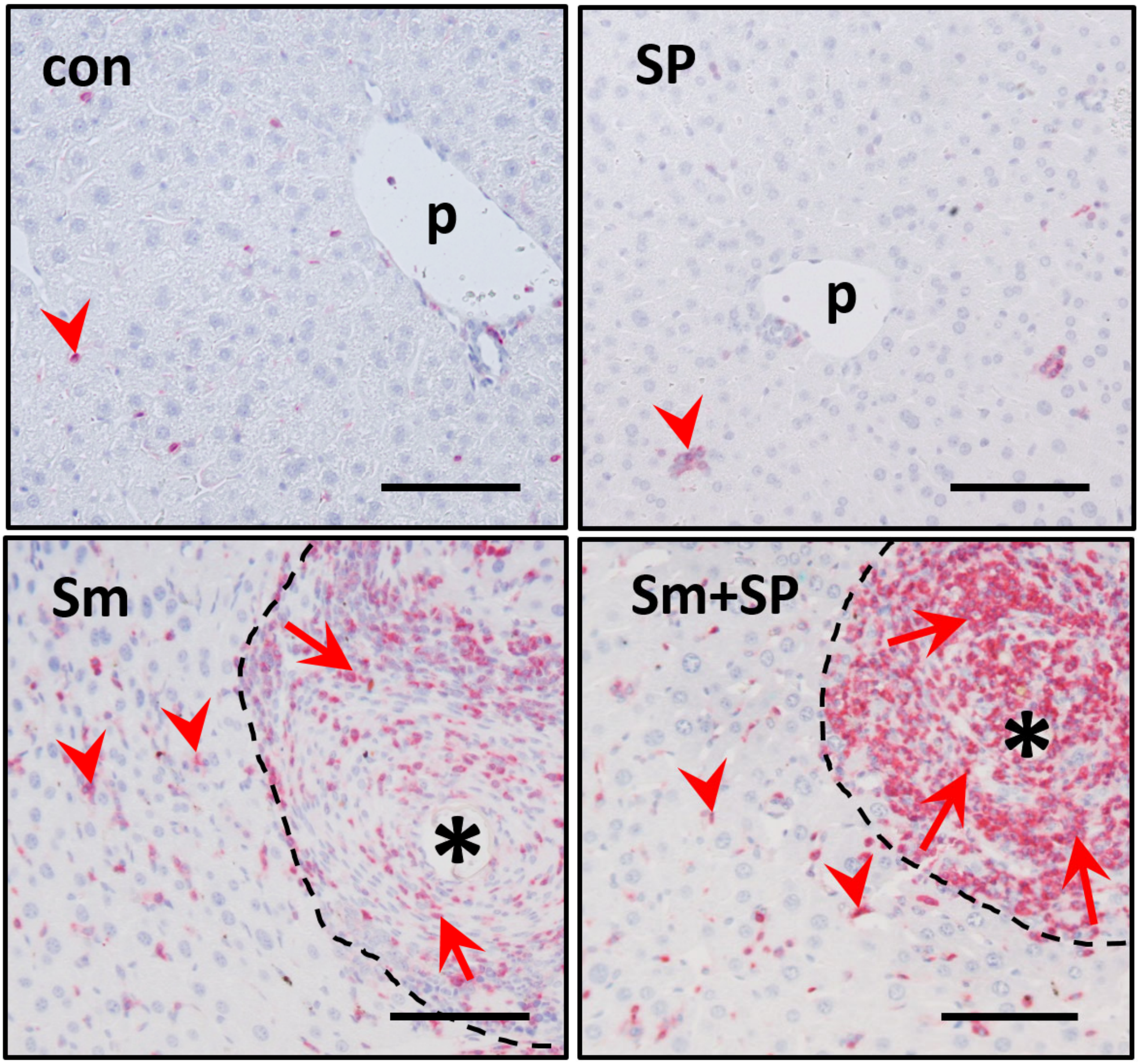

SFig. 11: Enlarged Figure 3A. CD45 immunostaining visualized an immense hepatic infiltration of CD45+ leukocytes, especially in the granulomas (arrows) of inhibitor-treated animals, but also into the parenchyma (arrowheads). P: portal field, *: S. mansoni egg, bars: 100µm.

SFig. 12

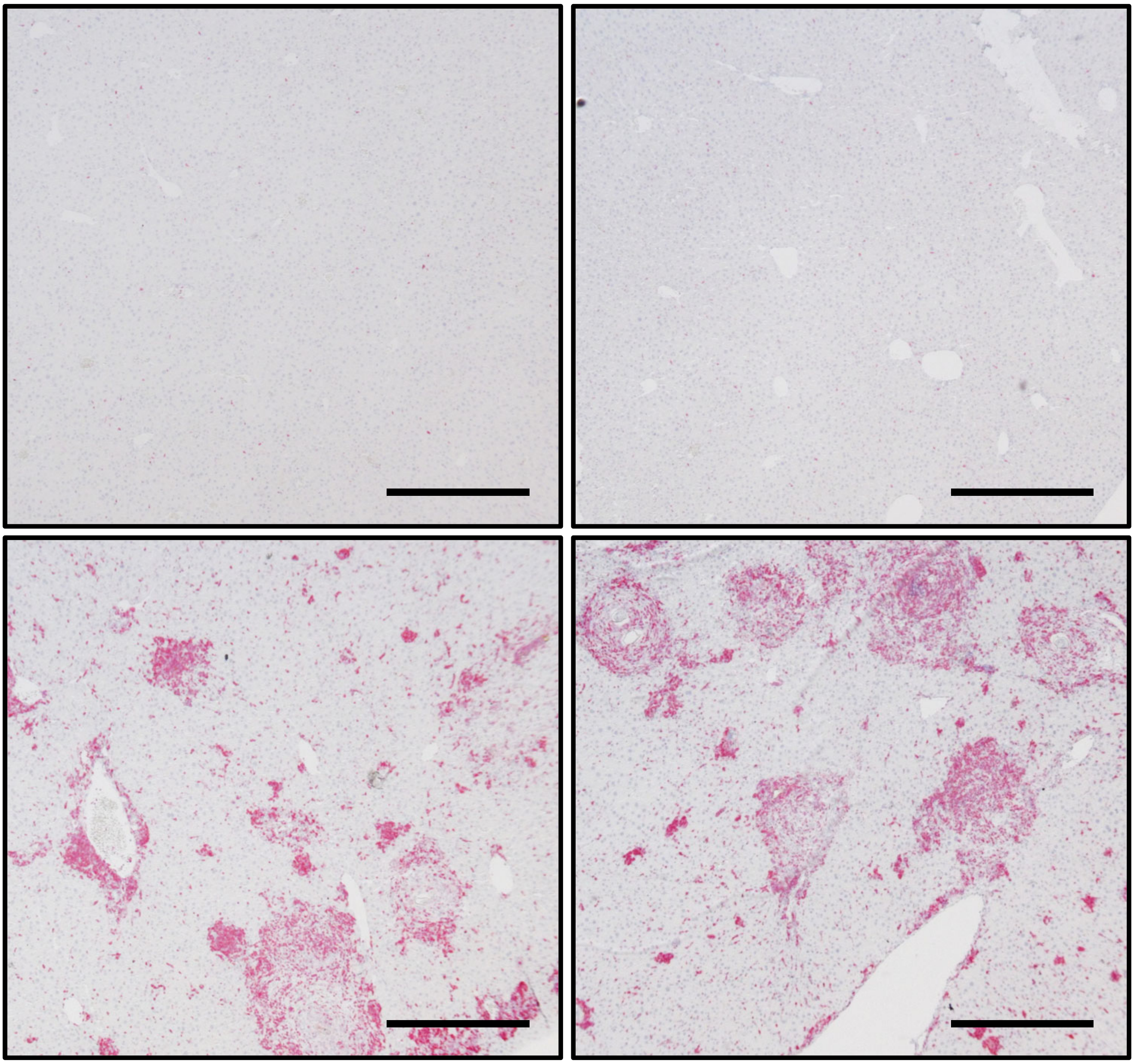

SFig. 12: Images of CD45 immunostainings with 50x magnification. CD45 immunostaining visualized hepatic leukocyte infiltration in *S. mansoni*-infected mice. Bars: 500µm. Representative liver slices are shown.

SFig. 13

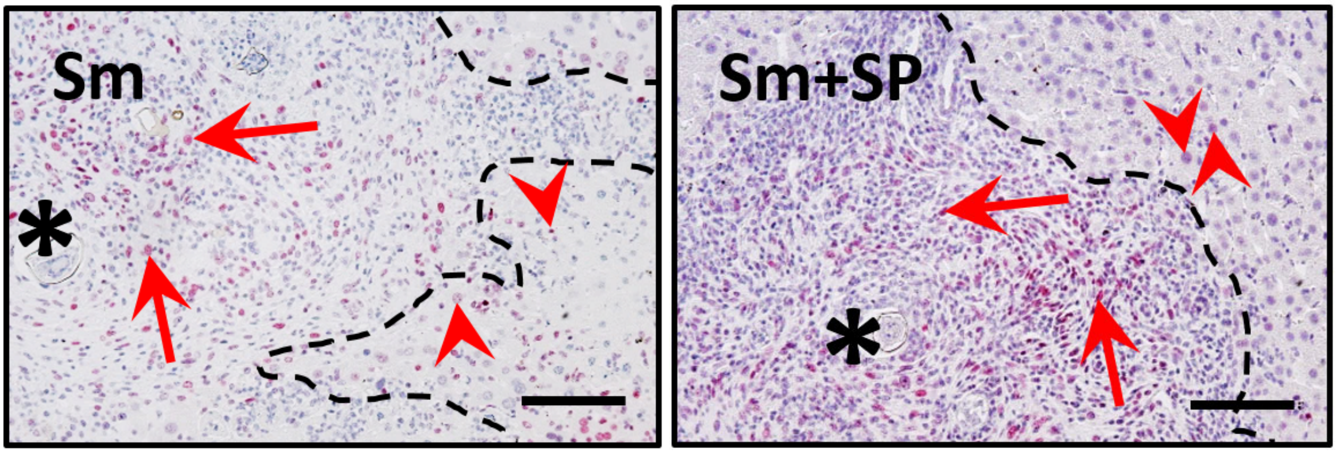

SFig. 13: Enlarged Figure 3F. P-STAT3 immunostaining revealed STAT3 activation inside the granulomas (arrows) but also in hepatocytes in direct vicinity (arrowheads). *: *S. mansoni* eggs, dashed line: border of granulomas, bars: 100 µm.

SFig. 14

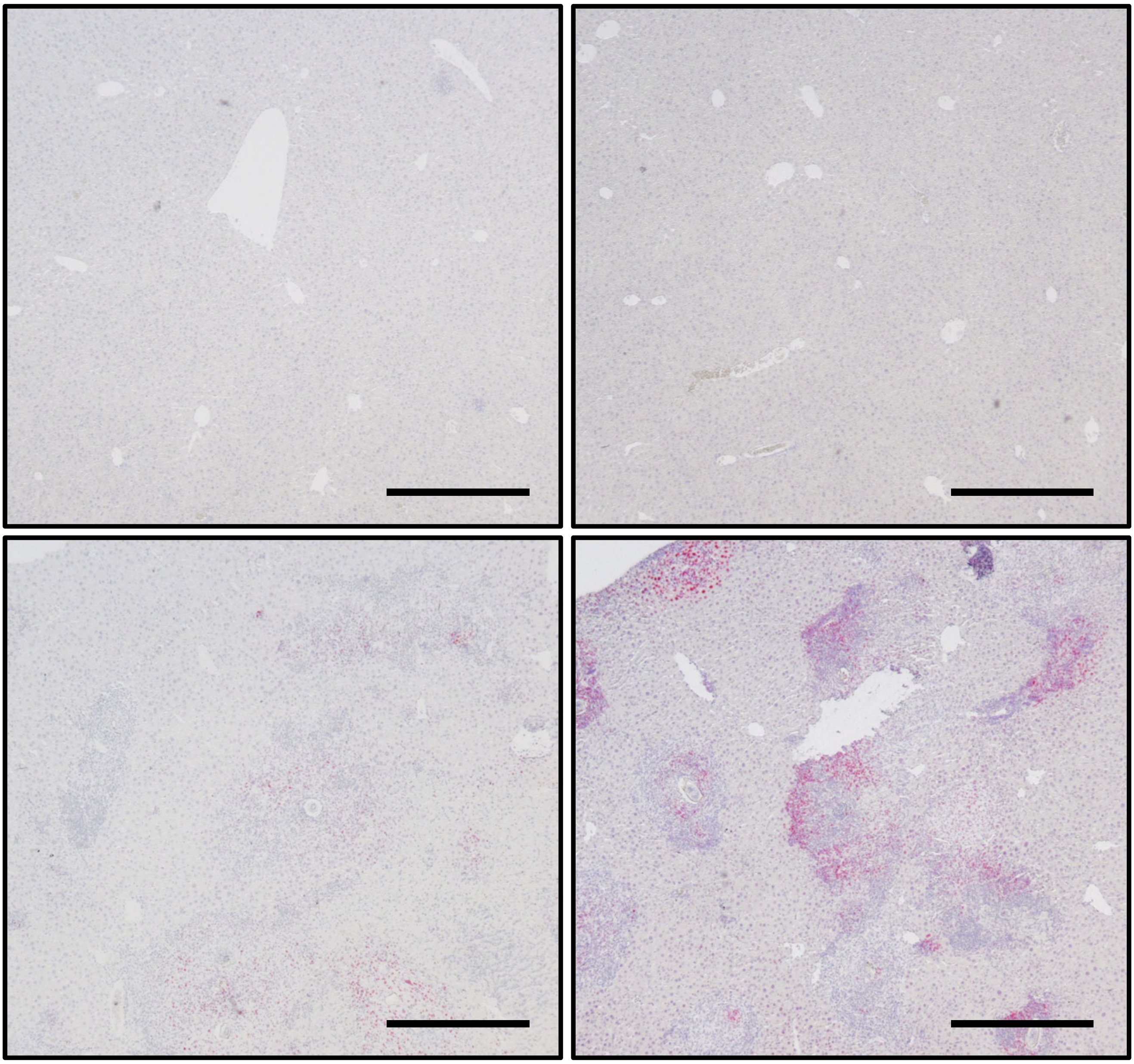

SFig. 14: Images of pSTAT3 immunostainings with 50x magnification. pSTAT3 immunostaining STAT3 activation inside the granulomas but also in hepatocytes in direct vicinity. Bars: 500µm. Representative liver slices are shown.

SFig. 15

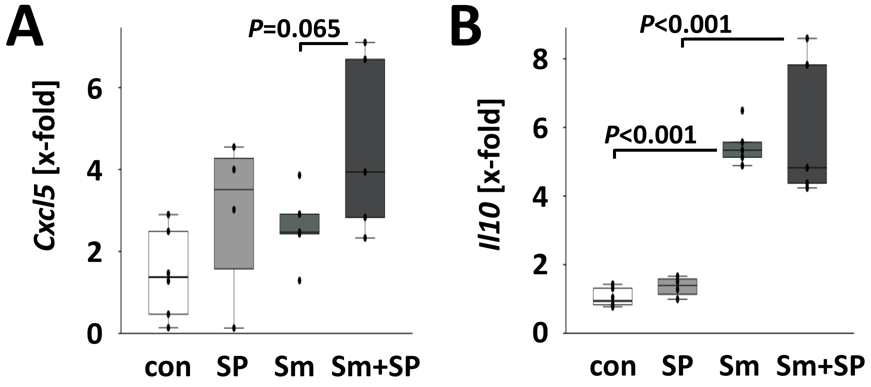

SFig. 15: Transcriptional levels of *Cxcl5* and *Il10*.

SFig. 16

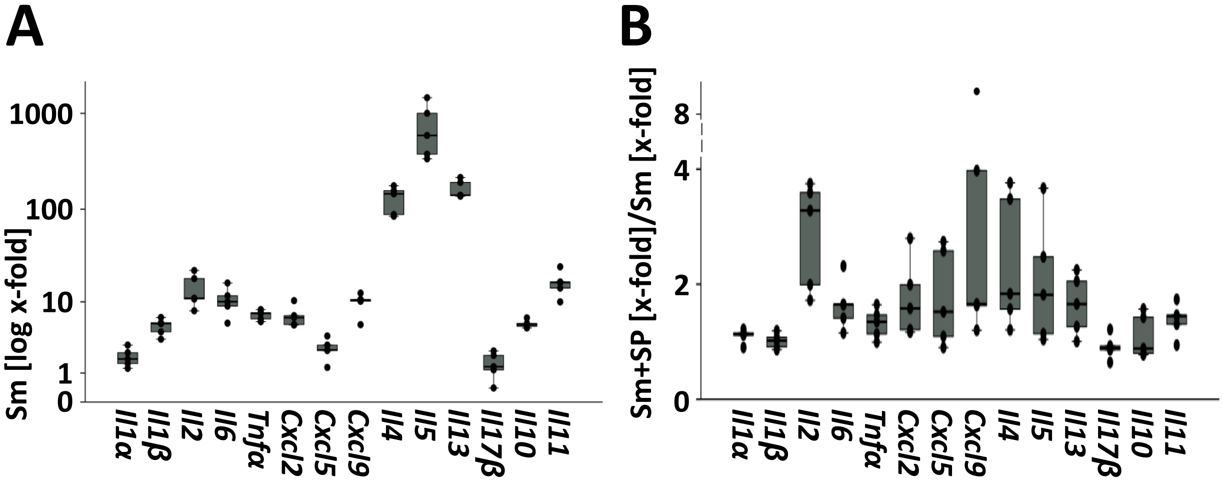

SFig. 16: *S. mansoni* and SP600125-regulated cytokine expression. (A) *S. mansoni*-induced cytokines. (B) Additional influence of SP600125.

SFig. 17

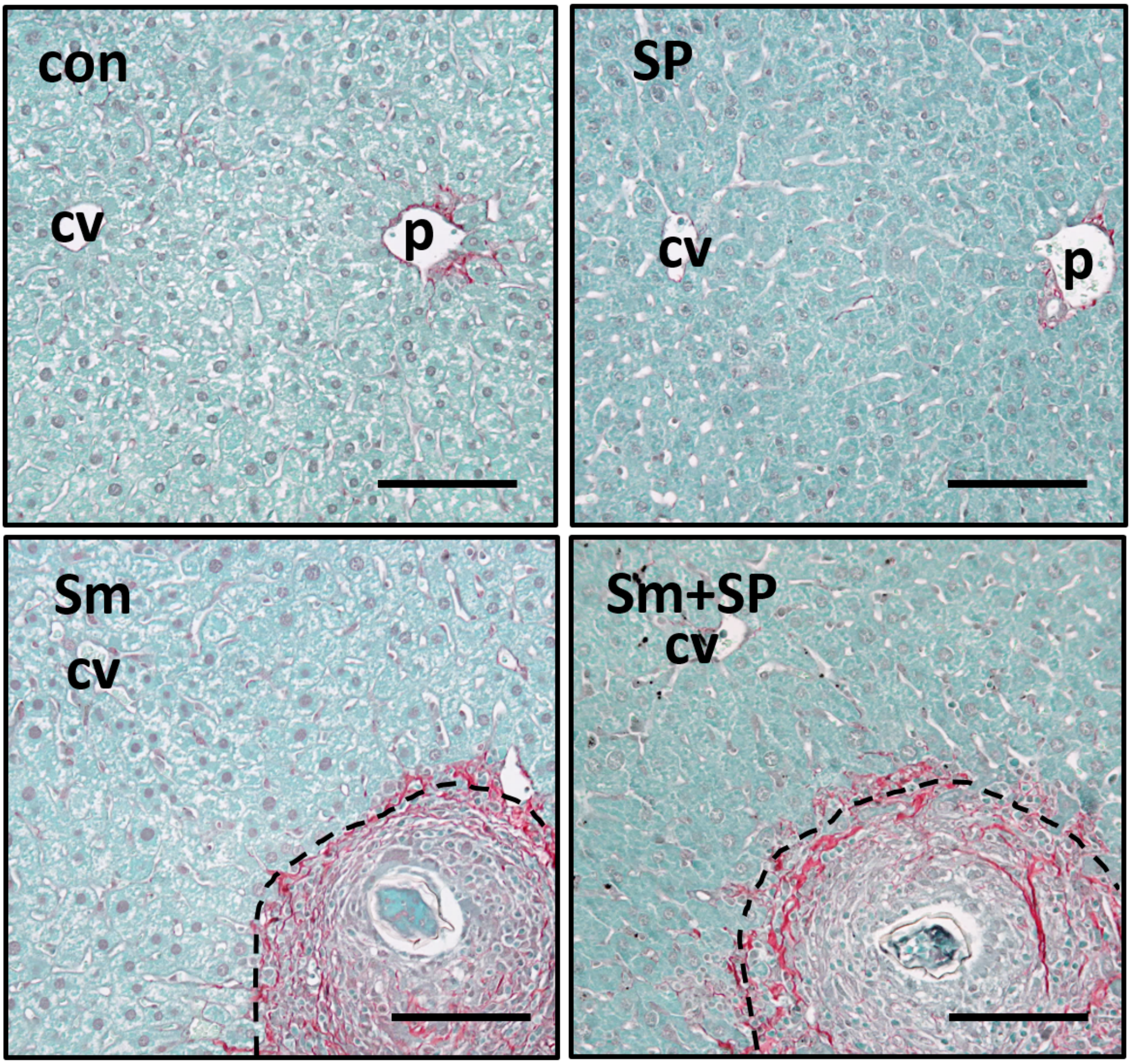

SFig. 17: Enlarged Figure 5A. Sirius Red-staining visualized the hepatic distribution of fibrillary collagens in red. Dashed line: border of granulomas, bars: 100µm.

SFig. 18

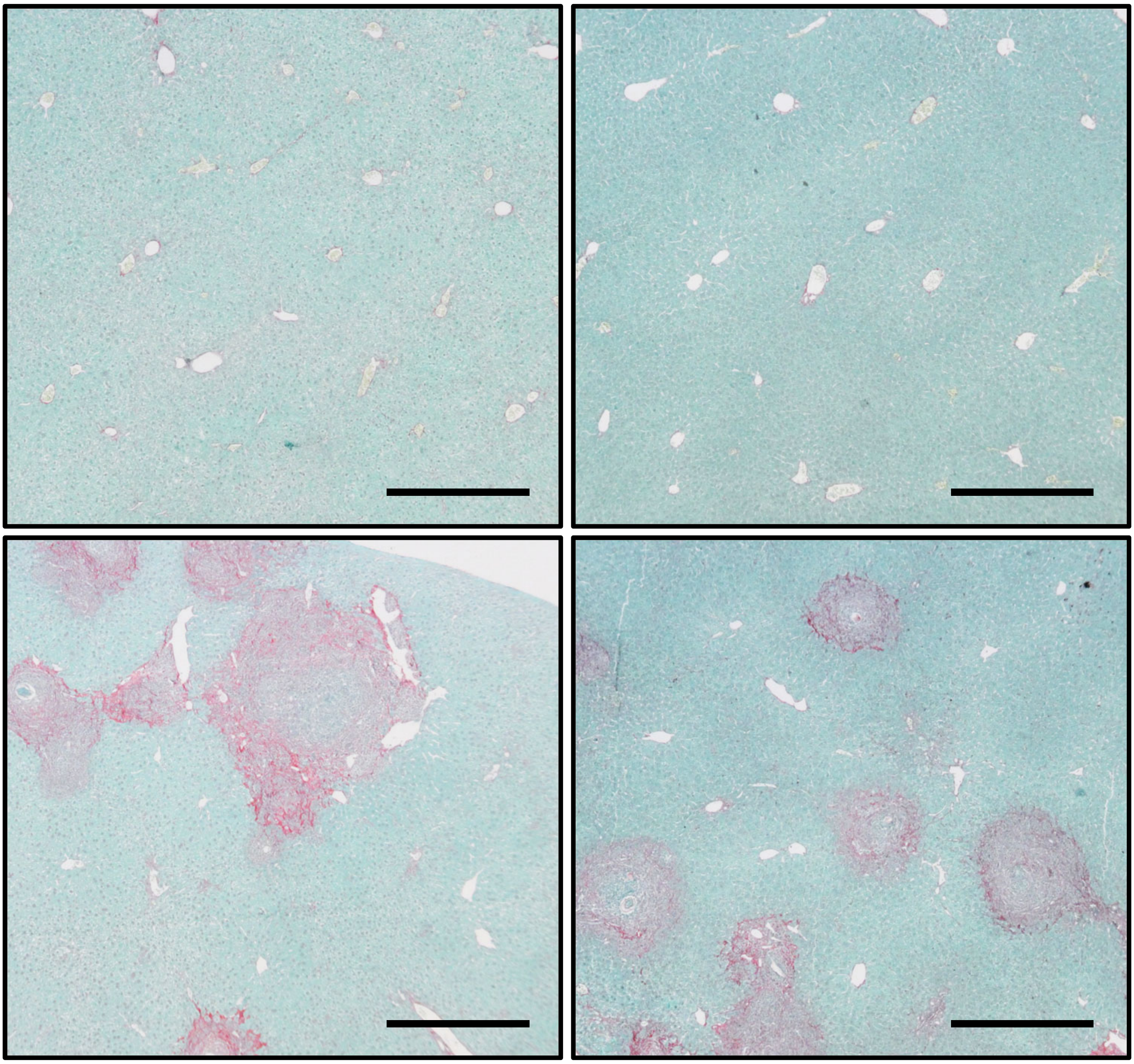

SFig. 18: Images of Sirius Red stained liver slices with 50x magnification. Sirius Red-staining visualized the hepatic distribution of fibrillary collagens in red. Bars: 500µm. Representative liver slices are shown.

SFig. 19

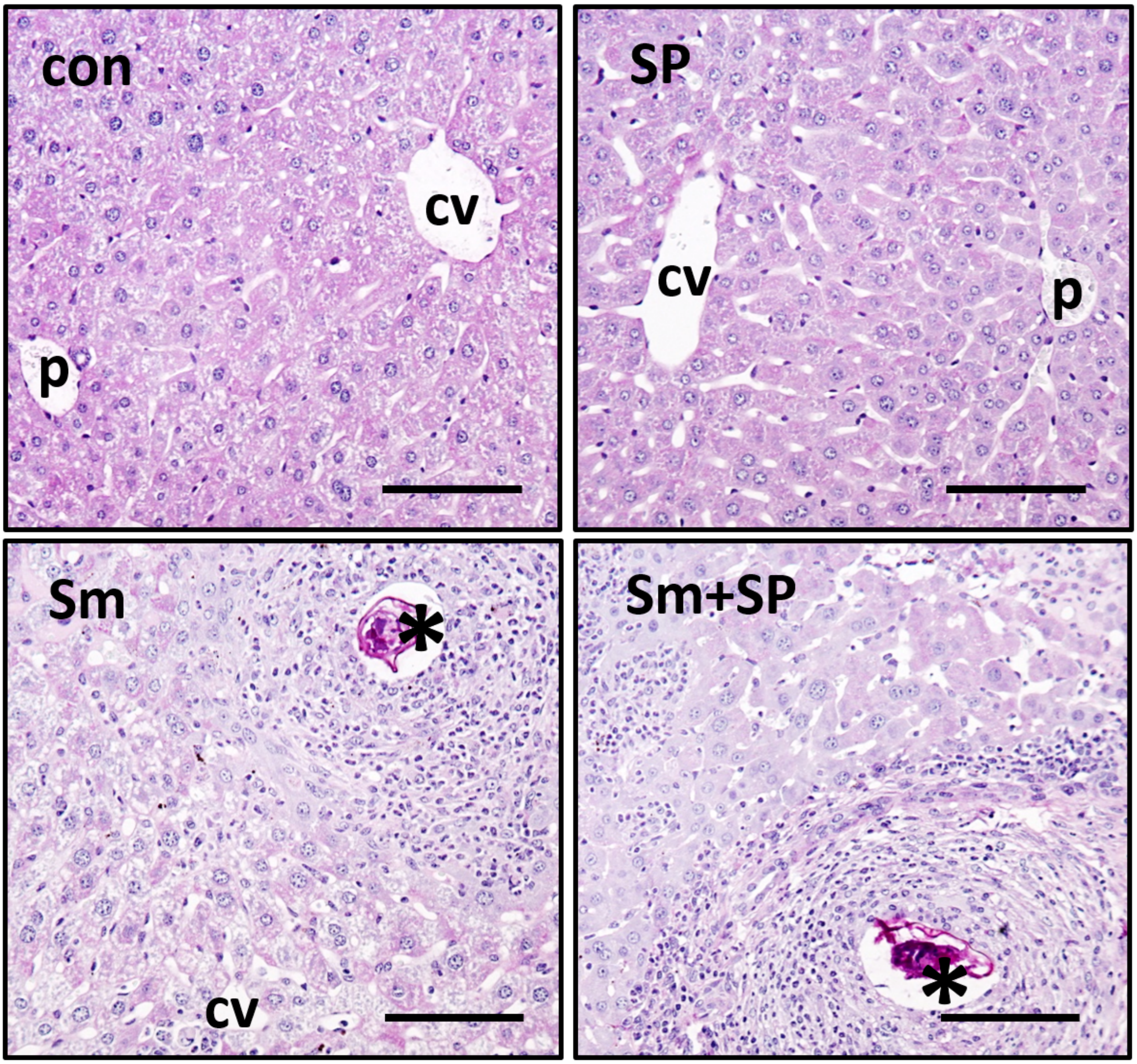

SFig. 19: Enlarged Figure 6A. PAS staining visualized glycogen exhaustion in the parenchyma and glycogen enrichment in the eggs of livers of *S. mansoni*-infected mice. Dashed line: border of granuloma, bars: 100µm, *: eggs.

SFig. 20

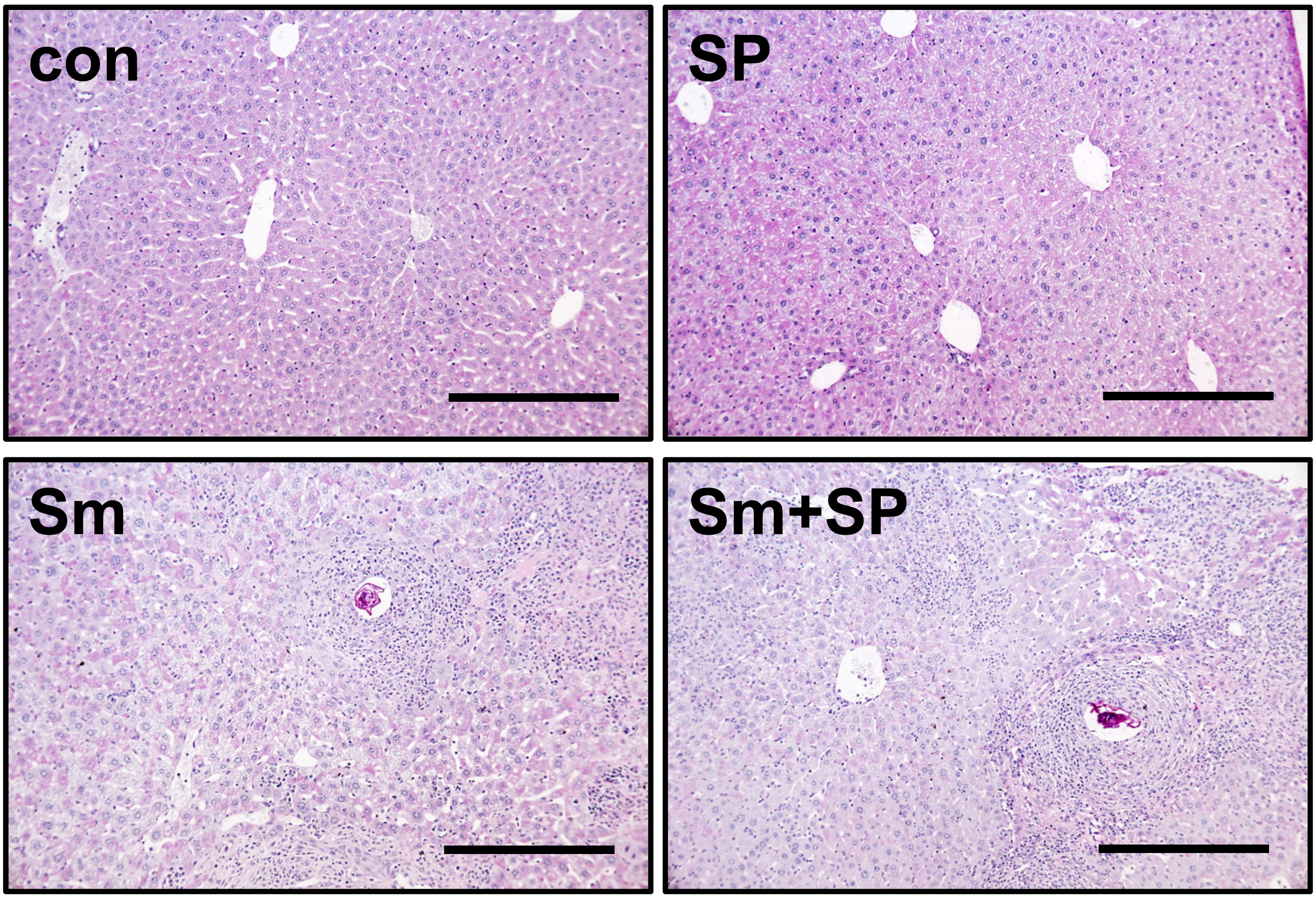

SFig. 20: Overview images of PAS-stained liver slices. PAS staining visualized glycogen exhaustion in the parenchyma and glycogen enrichment in the eggs of livers of *S. mansoni*-infected mice. Bars: 250µm. Representative liver slices are shown.

SFig. 21

A

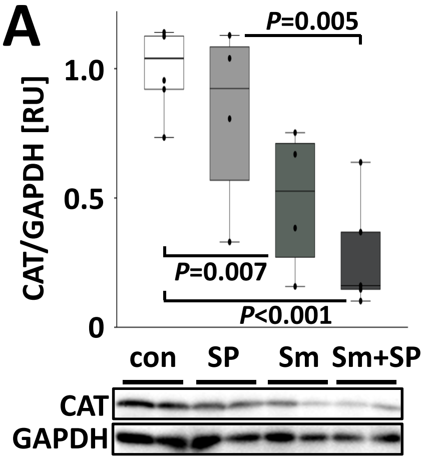

SFig. 21: Regulation of oxidative stress marker catalase: Western blotting demonstrated a reduced hepatic expression of catalase (CAT) in *S. mansoni*-infected mice. The decrease in CAT expression is tendentially stronger in *S. mansoni*-infected + SP600125-treated mice. White bars: uninfected control mice (con n=6), light grey bars: SP600125-treated mice (SP n=4) grey bars: *S. mansoni*-infected mice (Sm n=4) and dark grey bars: *S. mansoni*-infected and SP600125 treated (Sm+SP n=5). Two technical replicates. The indicated *p*-values were calculated by ANOVA and post hoc pairwise comparison of groups using Fisher’s LSD.

Supplementary Table 1:

gene	sense	antisense	manufact.
aSMA	data not available		Qiagen
CD45	5'-GAT GTC AGT TGG ACA ACC TTC G-3'	5'-GAT CAG GTT TAG ATG CAG GCT-3'	Microsynth
Col1A1	5'-GCT CCT CTT AGG GGC CAC T-3'	5'-CCA CGT CTC ACC ATT GGG G-3'	Microsynth
Col3A1	5'-GGA GAA AAT GGG AAA CCA GGT G-3'	5'-G AAC CAC CTT CAC CCT TAT CTC-3'	Microsynth
CXCL2	5'-GAC TTC AAG AAC ATC CAG AGC-3'	5'-TCC TTT CCA GGT CAG TTA GC-3'	Microsynth
CXCL5	5'-GCA TTT CTG TTG CTG TTC ACG CTG-3'	5'-CCT CCT TCT GGT TTT TCA GTT TAG C-3'	Microsynth
CXCL9	5'-TCC TTT TGG GCA TCA TCT TC-3'	5'-TTC CCC CTC TTT TGC TTT TT-3'	Microsynth
FASN	5‘-GTG AGT CTA TCC TGC GCT CC-3‘	5‘ -CGT CAG GTT TCA GTC CCA CA- 3‘	Microsynth
G6pdh	5‘-TCC TAC CAT CTG GTG GCT GT-3‘	5‘ -GCA AAG AAC TCC TCC AGC TT- 3‘	Microsynth
GAPDH	5'-TGA GGA CCA GGT TGT CTC CT-3'	5'-TGT GAG GGA GAT GCT CAG TG-3'	Microsynth
IL-1a	5'-CGA AGA CTA CAG TTC TGC CAT T-3'	5'-GAC GTT TCA GAG GTT CTC AGA G-3'	Microsynth
IL-1b	5‘-TGA CAG TGA TGA GAA TGA CCT G-3‘	5‘-CGG GAA AGA CAC AGG TAG CT-3‘	Microsynth
IL-2	5‘ –CAC TGA CAC TTG TGC TCC TT- 3‘	5‘ –GAA AGT CCA CCA CAG TTG CT- 3‘	Microsynth
IL-4	5'-GGT CTC AAC CCC CAG CTA GT-3'	5'-GCC GAT GAT CTC TCT CAA GTG AT-3'	Microsynth
IL-5	5‘ –TTG ACA AGC AAT GAG ACG ATG AG- 3‘	5‘ –ACC AGT TTG AGG CCA GCC TGC G- 3‘	Microsynth
IL-6	5'-TCC AGT TGC CTT CTT GGG AC-3'	5'-GTA CTC CAG AAG ACC AGA GG-3'	Microsynth
IL-6Ra	5'CCT GAG ACT CAA GCA GAA ATG G-3'	5'-AGA AGG AAG GTC GGC TTC AGT-3'	Microsynth
IL-6st	5'-CCG TGT GGT TAC ATC TAC CCT-3'	5'-CGT GGT TCT GTT GAT GAC AGT G-3'	Microsynth
IL-10	5'-CCC ATT CCT CGT CAC GAT CTC-3'	5'-TCA GAC TGG TTT GGG ATA GGT TT-3'	Microsynth
IL-11	5'-TGT TCT CCT AAC CCG ATC CCT-3'	5'-CAG GAA GCT GCA AAG ATC CCA-3'	Microsynth
IL-13	5'-CCT GGC TCT TGC TTG CCT T-3'	5'-GGT CTT GTG TGA TGT TGC TCA-3'	Microsynth
IL-17b	5'-GAG TAA AGC CCT ACG CTC GAA-3'	5'-CTC CTC TTG TTG GAC AAC CAC-3'	Microsynth
STAT6	5'-CTC TGT GGG GCC TAA TTT CCA-3'	5'-CAT CTG AAC CGA CCA GGA ACT-3'	Microsynth
TNFa	5‘-GCC CAC GTC GTA GCA AAC CAC-3‘	5‘-GCA GGG GCT CTT GAC GGC AG-3‘	Microsynth
